# Graded Nodal/Activin Signaling Titrates Conversion of Quantitative Phospho-Smad2 Levels into Qualitative Embryonic Stem Cell Fate Decisions

**DOI:** 10.1371/journal.pgen.1002130

**Published:** 2011-06-23

**Authors:** Kian Leong Lee, Sandy Keat Lim, Yuriy Lvovich Orlov, Le Yau Yit, Henry Yang, Lay Teng Ang, Lorenz Poellinger, Bing Lim

**Affiliations:** 1 Cancer Science Institute of Singapore, National University of Singapore, Singapore, Singapore; 2 Genome Institute of Singapore, Agency for Science, Technology, and Research, Singapore, Singapore; 3 Duke-NUS Graduate Medical School, National University of Singapore, Singapore, Singapore; 4 NUS Graduate School for Integrative Sciences and Engineering, National University of Singapore, Singapore, Singapore; 5 Institute of Cytology and Genetics, Siberian Branch of the Russian Academy of Sciences, Novosibirsk, Russia; 6 Singapore Immunology Network, Agency for Science, Technology, and Research, Singapore, Singapore; 7 Department of Cell and Molecular Biology, Karolinska Institutet, Stockholm, Sweden; 8 Beth Israel Deaconess Medical Centre, Harvard Medical School, Boston, Massachusetts, United States of America; Ludwig Institute for Cancer Research and University of California San Diego, United States of America

## Abstract

Nodal and Activin are morphogens of the TGFbeta superfamily of signaling molecules that direct differential cell fate decisions in a dose- and distance-dependent manner. During early embryonic development the Nodal/Activin pathway is responsible for the specification of mesoderm, endoderm, node, and mesendoderm. In contradiction to this drive towards cellular differentiation, the pathway also plays important roles in the maintenance of self-renewal and pluripotency in embryonic and epiblast stem cells. The molecular basis behind stem cell interpretation of Nodal/Activin signaling gradients and the undertaking of disparate cell fate decisions remains poorly understood. Here, we show that any perturbation of endogenous signaling levels in mouse embryonic stem cells leads to their exit from self-renewal towards divergent differentiation programs. Increasing Nodal signals above basal levels by direct stimulation with Activin promotes differentiation towards the mesendodermal lineages while repression of signaling with the specific Nodal/Activin receptor inhibitor SB431542 induces trophectodermal differentiation. To address how quantitative Nodal/Activin signals are translated qualitatively into distinct cell fates decisions, we performed chromatin immunoprecipitation of phospho-Smad2, the primary downstream transcriptional factor of the Nodal/Activin pathway, followed by massively parallel sequencing, and show that phospho-Smad2 binds to and regulates distinct subsets of target genes in a dose-dependent manner. Crucially, Nodal/Activin signaling directly controls the *Oct4* master regulator of pluripotency by graded phospho-Smad2 binding in the promoter region. Hence stem cells interpret and carry out differential Nodal/Activin signaling instructions via a corresponding gradient of Smad2 phosphorylation that selectively titrates self-renewal against alternative differentiation programs by direct regulation of distinct target gene subsets and *Oct4* expression.

## Author Summary

Nodal and Activin are extracellular signaling molecules that diffuse from the source of secretion and induce recipient stem cells to become new cell types according to a concentration gradient. In the early embryo, they are important for the specification of tissues at the correct place and time, but paradoxically they drive the opposite function in embryonic and epiblast stem cells where they maintain the stem cell state instead of promoting differentiation. The molecular basis of how the level of signaling determines stem cell fate decisions remains poorly understood. We found that Smad2, the main transcription factor of the Nodal/Activin pathway was phosphorylated according to the level of signaling. By mapping where phospho-Smad2 binds in the embryonic stem cell genome and how this affects transcription of the associated target genes, we show that phospho-Smad2 can recruit and regulate different sets of target gene depending on the signaling level. Moreover, phospho-Smad2 also directly regulates *Oct4*, a master gene controlling the stem cell state thereby reconciling the opposing functions of the Nodal/Activin pathway in differentiation versus self-renewal programs. The pathway can mediate the exit from self-renewal via *Oct4* and simultaneously drives differentiation towards particular lineages by recruiting the relevant gene subsets for this purpose.

## Introduction

Morphogens are secreted signaling molecules that orchestrate the spatial distribution and sequence of cellular differentiation events throughout embryonic development. The specific cell types, their localization and order of induction from recipient stem cell populations are determined by the concentration gradient of morphogens diffusing from the source of secretion. Previous studies have proposed some of the models by which morphogen gradients are initiated, established and stabilized including the level of receptor occupancy, positive/negative feedback and feed forward mechanisms [1]–[3]. However, little is understood about the transcriptional mechanisms responding to variable receptor activation and how they permit pluripotent stem cells to interpret signaling levels and direct the appropriate differentiation programs during mammalian development.

Nodal and Activin are morphogens of the TGFβ superfamily of signaling molecules. In *Xenopus* embryos, Activin is a potent concentration-dependent inducer of mesoderm, mesendoderm and endoderm in animal cap cells [2], [4], [5]. Nodal has also been shown to be a classical morphogen in zebrafish where it functions in a concentration gradient independently of any relaying mechanisms [6]. In the early mouse embryo, mutations that perturb the level of Nodal/Activin signaling show that the pathway plays crucial roles in the induction of the primitive streak/mesoderm, mammalian organizer (node), mesendoderm and endoderm during the establishment of the anterior-posterior axis [7]–[11]. In contrast to *in vivo* evidence that Nodal/Activin signaling predominantly promotes differentiation events, the pathway also paradoxically has important roles in the maintenance of self-renewal and pluripotency. Indeed Activin A is frequently used directly in culture for the continued propagation and expansion of human embryonic and mouse epiblast stem cells [12]–[15].

The signaling level of the Nodal/Activin pathway is determined by the overall activity of its components many of which have been identified. Both the Nodal and Activin ligands bind to the same type I/II serine-threonine receptor kinase complexes consisting of ActRIIA/B and Alk4/5/7 respectively in the mouse [16]. Nodal requires the cofactors Cripto/Criptic for receptor activation as opposed to Activin that can bind directly to the receptors and is inhibited by Cripto [17]–. Upon ligand docking, the Type I receptors phosphorylate the downstream signal transducers Smad2 and Smad3 (Smad2/3) which form hetero- or homodimers and trimers [20]. Both Smad2/3 are also phosphorylated by crosstalk with EGF/ERK/MAPK signaling [21]–[23] but only the serine residues of the SSXS motif on the extreme carboxy terminus are specifically phosphorylated by Nodal/Activin/TGFbeta signaling. This phosphorylation is important for the translocation of Smad2/3 to the nucleus in association with Smad4 [24], [25] where the complex recruits a number of transcription factors including FoxHI, p53, β-catenin and Jun/Fos for the direct regulation of target genes [20]. Specificity of the Smads for their direct target genes is partly conferred by a DNA domain in the MH1 region to the Smad-binding DNA element (SBE) consisting of a basic CAGA sequence or its complement [26]. The other partner transcription factors within the complex are required for additional target gene affinity and specificity.

While Smad2/3 share more than 90% protein homology, they are not functionally equivalent. Full-length Smad2 differs from Smad3 as the presence of an inhibitory domain in the MH1 region prevents direct DNA binding while Smad3 can bind directly to SBE boxes [27]. However, an alternatively spliced variant of Smad2 that lacks the inhibitory domain can bind DNA directly and has been shown to be the isoform that accounts for all developmental Smad2 functions *in vivo* [28]. The developmental roles of Smad2/3 are also disparate. *Smad2* knockout mouse embryos fail to form mesoderm and endoderm due to defects in primitive streak specification after implantation at 6.5 dpc [29] closely phenocopying *Nodal* mutants [10]. In contrast, *Smad3* mutant mice are born alive and are fertile but develop chronic intestinal inflammation leading to colorectal cancer [30]. This suggests that Smad2 is the primary transcriptional mediator of early developmental events while Smad3 is involved in immune function and possibly acts as a tumor suppressor postnatally.

Our focus here is to clarify how mechanistically different levels of Nodal/Activin signaling lead to different embryonic stem (ES) cell fate decisions. ES cells were differentiated using three different quanta levels of Nodal/Activin signaling. We showed that ES cells are able to arbitrate between three distinct cell fate decisions. Maintenance of endogenous Nodal/Activin signaling is required for self-renewal of ES cells where any perturbation leads to an exit from self-renewal and pluripotency programs towards mesendoderm induction at high signaling and trophectoderm differentiation at low signaling.

One obvious question to resolve is whether different levels of Nodal/Activin signaling recruit different sets of genes. While genome wide transcriptome studies have suggested possible Nodal/Activin targets, the identity of many transcriptional targets directly regulated by Smad2/3 remains unknown. One ChIP-chip study to date has been performed to address endogenous Smad2/3 binding in transformed human keratinocytes [31] while none have been carried out in the context of stem cell fate decisions, graded Nodal/Activin signaling or examining beyond promoter regions. Here we performed quantitative chromatin immunoprecipitation (ChIP) of phospho-Smad2 (pSmad2) during graded Nodal/Activin signaling followed by massively parallel sequencing (ChIP-Seq) covering the full extent of pSmad2 binding to the ES cell genome including 5′/3′ UTRs, exons/introns and gene deserts. PSmad2 binding and regulation of direct target gene expression does not vary uniformly across the genome but changes in both a qualitative and quantitative manner with different signaling levels. Some targets are up- or downregulated proportionate to the activity of the Nodal/Activin pathway. However, separate subsets of target genes are regulated only during high or low signaling conditions. The downstream consequences of this is differential expression of the target genes that combine dose-dependent genes with different subsets of genes activated or repressed specifically for each signaling level. Thus ES cells carry out alternative cell fate decisions via the recruitment of target gene subsets in a pSmad2 dose-dependent manner.

To reconcile some of the conflicting functions of Nodal/Activin signaling in self-renewal and pluripotency versus differentiation cell fate decisions, we examined the regulation of the *Oct4* pluripotency and self-renewal master gene. *Oct4* was directly regulated by pSmad2 binding in the promoter region independent of all other cis regulatory elements. Consistent with the modulation of pSmad2 binding, both endogenous mRNA and protein levels of Oct4 were also repressed by inhibition of Nodal/Activin signaling. Hence pSmad2 is a direct upstream regulator of *Oct4* transcription where it permits an exit from maintenance of the stem cell state towards mesendoderm or trophectoderm differentiation programs as specified by the signaling level.

In conclusion, the molecular switching of binding locations and target genes by pSmad2 across the ES cell genome in a dose-dependent manner provides a mechanism for the shift in the balance between maintenance of the stem cell state and the opposing induction of differentiation. Key signaling pathways have been predominantly studied in a binary context where they are either present or absent in a biological system. This view has only been able to account for some of their many and often conflicting roles. Our findings challenge this view and support multi-level signaling in stem cells where different signaling strengths can engender different cell fate decisions reflective of the *in vivo* development of embryos directed not just by Nodal/Activin signaling but possibly Hedgehog, FGF, Wnt and other morphogen pathways.

## Results

### The Peak of Transcriptional Activity Induced by Graded Nodal/Activin Signaling Occurs at 18 Hours

The direct cellular function of the Nodal/Activin pathway notably of the downstream components Smad2/3/4 is for the regulation of transcription. To address the relation between graded signaling and how they affect transcription, we quantified the changes in expression of known target genes under different signaling levels in chemically defined KSR media conditions. Pluripotent mouse embryonic stem (ES) cells were used to assess the mechanism of morphogen activity as they can differentiate into all tissue types of the adult and express all components of the pathway permitting response to manipulated Nodal/Activin signaling.

Some of the known target genes include *Pitx2* and *Lefty2* which are responsible for the establishment of left-right asymmetry during early embryogenesis, a key developmental role of Nodal/Activin signaling [32]. In addition, both *Lefty2* and *Smad7* function as inhibitors of the pathway in a negative feedback mechanism for the attenuation of Nodal/Activin signaling strength [33], [34]. Although direct Smad2/3 binding and regulation of the *Pitx2* and *Lefty2* genes have not yet been demonstrated, *in vivo* reporter assays suggest that specific enhancers are responsive to Nodal/Activin signaling and are active only on the left side of the embryo [35], [36]. Moreover, these enhancers have been shown to contain FoxH1 binding sites, a known key transcriptional copartner of Smad2/3. *Smad7* has been shown to be a direct target of Smad2/3/4 binding in the promoter region by gel shift assays [37], [38] and it antagonizes the interaction of Smad2/3 with the Type I kinase receptors [39] during negative feedback.

**Figure 1:**
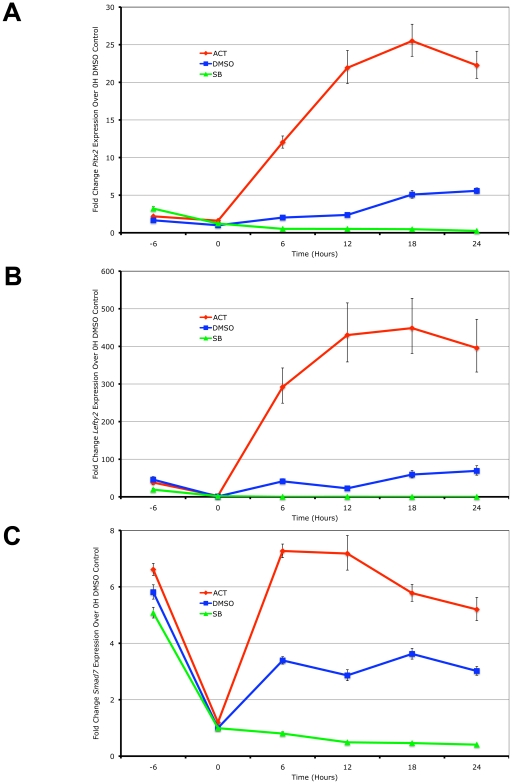
*Pitx2*, *Lefty2*, and *Smad7* Are Transcriptional Targets of Graded Nodal/Activin Signaling. Real-time PCR quantification of changes in mRNA levels for (A) *Pitx2*, (B) *Lefty2* and (C) *Smad7* in mouse ES cells during a 0 to 24 hours time course under graded Nodal/Activin signaling conditions. High signaling was induced by direct treatment with 25 ng/ml Activin (red), low signaling with 10 uM of SB431542 inhibitor (green) and a control treatment with 1/5000 dilution of DMSO carrier (blue) was carried out following pretreatment of all cells for 6 hours in chemically defined KSR media with 10 µM SB (−6 to 0 hours). β-actin was used as a housekeeping control and error bars show s.e.m for n = 3 replicates.

Using real-time PCR quantitation, the expression of the 3 target genes was examined in the ES cells following the induction of high signaling by direct treatment with Activin in a time-course. In the reciprocal experiment, the small chemical inihibitor SB-431542 that specifically prevents the kinase domains of the Type I kinase receptors from phosphorylating Smad2/3 [40] was used to generate low Nodal/Activin signaling conditions. *Pitx2*, *Lefty2* and *Smad7* were up- and downregulated in direct correlation with the level of signaling under chemically defined conditions compared to the DMSO carrier control representing endogenous or medium signaling (Figure 1). Over the course of 24 hours, the maximum expression of *Pitx2* and *Lefty2* occurred at 18 hours (Figure 1A and 1B) while that of *Smad7* (Figure 1C) occurred earlier at 12 hours. We therefore conclude based on these known target genes that Nodal/Activin signal transduction and its effects on transcription require up to 18 hours to fully develop and any earlier time points result in weaker inductions.

We further confirmed that *Pitx2*, *Lefty2* and *Smad7* are direct targets of the Nodal/Activin pathway by conducting chromatin immunoprecipitation of phosphorylated Smad2 in the ES cells under the same chemically defined conditions at 18 hours followed by quantification of the enriched genomic DNA fragments by real-time PCR using tiling primers (Figure S1 and Table S3). The antibody used for the pulldown was raised against the phosphorylated serines 465 and 467 on the carboxy-terminus of Smad2 that are specifically targeted by TGFbeta signaling and not by EGF/ERK/MAPK signaling. At 18 hours where there is maximum expression of the 3 target genes, there was also a robust divergence in the level of pSmad2 binding according to the signaling level for the enhancers of *Pitx2* and *Lefty2* (Figure S1A and S1B). Interestingly pSmad2 binding was invariant on the known TGFbeta response element of the *Smad7* promoter (Figure S1C). This suggested that Nodal/Activin target genes had different binding efficiencies for pSmad2 at each Nodal/Activin signaling level and this was not uniformly changed for all target genes.

In conclusion, we confirm that *Pitx2*, *Lefty2* and *Smad7* were direct targets of Nodal/Activin signaling and graded pSmad2 binding. Differential signaling sustained for 18 hours also leads to the maximum level of differential gene expression with clear changes in pSmad2 binding on the *Pitx2* and *Lefty2* genes.

### Graded Nodal/Activin Signaling Mediates 3 Distinct Cell Fate Decisions in ES Cells

Given the downstream changes in pSmad2 binding and transcription of the known direct target genes, we next addressed how extracellular signal levels are translated into intracellular levels of signal transduction. We hypothesized that this could be directly related to changes in pSmad2 levels in ES cells as a consequence of Type I receptor kinase activity. Hence ES cells subjected to differential morphogen signaling conditions may be able to produce different amounts of pSmad2 in cells generating a corresponding differential level of intracellular signaling that leads to differential transcription.

**Figure 2:**
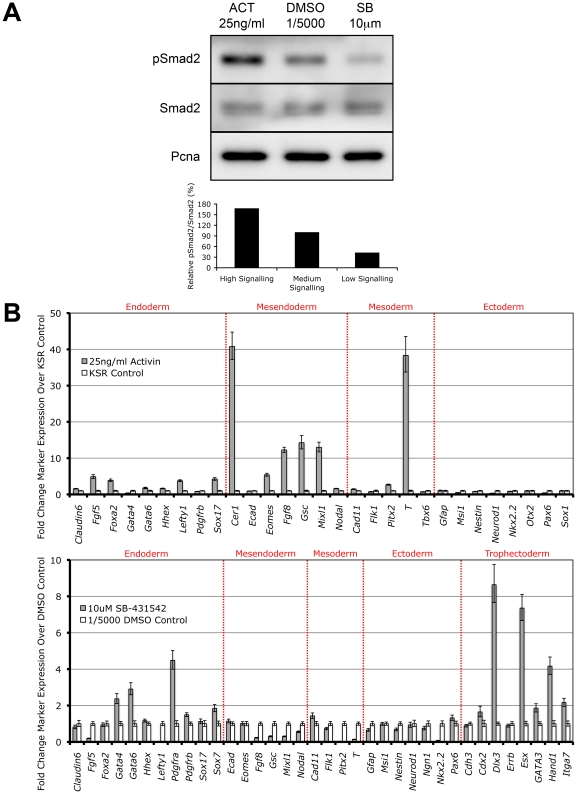
Smad2 Phosphorylation and Induction of Distinct Cell Fate Decisions Are Determined by Differential Nodal/Activin Signaling. (A) Western blot and densitometry quantitation of Smad2 phosphorylation in ES cells after 18 hours stimulation with Activin (ACT) and inhibition with SB-431542 (SB) compared to DMSO vehicle control at the indicated doses in chemically defined KSR media. The same blot was stripped and reprobed with total Smad2 and Pcna loading control antibodies. Secondary band in the total Smad2 blot corresponds to cross-reaction with total Smad3. Graph shows densitometry measurements of pSmad2 protein bands relative to total Smad2 in each treatment with the DMSO control at 100%. (B) Real-time PCR analysis of early cell fate markers in differentiated ES cells after 6 days treatment in ACT, DMSO and SB normalized to β-actin housekeeping control. Top panel shows induction of markers in ACT treatments expressed as fold change over the control KSR media. Bottom panel shows fold change in marker expression during SB treatment compared to KSR media supplemented with 1/5000 DMSO vehicle. Error bars show standard error of the mean (s.e.m) for n = 4 replicates.

It has recently been shown that overexpression of the constitutively active Alk4 type I kinase receptor is sufficient to drive phosphorylation of Smad2 independent of all other Nodal/Activin receptor complex components [41]. Here we show that direct treatments of the ES cells with Activin and the specific Type I receptor kinase inhibitor SB-431542 in chemically defined conditions also tightly regulates receptor complex activity and produces the phosphorylation of Smad2 in a signaling dependent manner (Figure 2A). During Activin stimulation (high signaling) for 18 hours, there is a defined 2-fold increase in pSmad2 levels while repression with 10 µM SB (low signaling) leads to a 2-fold decrease that is within the limits of physiological change compared to the DMSO vehicle control (equivalent of medium signaling). Differential signaling had no effect on the equilibrium of total Smad2 suggesting that only phosphorylation and not regulation of the total Smad2 population is mediated by Nodal/Activin signaling. Hence extracellular signaling levels are translated into an equivalent gradient of intracellular Smad2 phosphorylation in ES cells.

Subsequently we addressed the long-term consequences of increased and decreased signaling on ES cell fate decisions by examining how manipulation of the pathway recapitulates *in vivo* cell fate decisions by direct treatment with Activin or SB for 6 days. Analysis of a broad range of early cell fate markers (Figure 2B) shows that enhanced Nodal/Activin signaling promotes mesendoderm differentiation in ES cells with strong upregulation of mesendodermal lineage genes including *Gsc*, *Mixl*, *Eomes* and *Fgf8*. The marker for mesoderm, *Brachyury* (*T*), was also strongly induced although this was not reflected by the other mesodermal markers such as *Flk1* and *Tbx6*. This was consistent with the finding that *T* is also co-expressed in mesendoderm *in vivo* at the anterior primitive streak [42]. Taken together, this suggests that high signaling induced by Activin predominantly drives mesendoderm differentiation.

Conversely, inhibition of the pathway with SB (Figure 2B) led to the upregulation of trophectoderm specific markers including *Dlx3*, *Esx* and *Hand1* and a less significant induction of extraembryonic primitive endoderm markers such as *Gata4/6* and *Pdgfra*. Similar results were obtained when the ES cells were treated with recombinant Lefty1 protein for the same period of time (data not shown) suggesting that the trophectoderm induction was specific to low Nodal/Activin signaling. Interestingly there was no induction of mesendodermal markers as in the Activin treatment and instead some of these such as *Gsc*, *Mixl* and *Fgf8* were strongly downregulated. Together, these results suggest that perturbation of the level of Nodal/Activin signaling and consequently endogenous Smad2 phosphorylation led to an exit from self-renewal in ES cells towards highly divergent cell fate decisions of either mesendoderm or trophectoderm differentiation.

To confirm these results, fluorescent immunostaining was carried out to assess the protein markers of trophectodermal and mesendodermal lineages (Figure S2) after differentiation in serum containing media. The cell fates obtained under these conditions are similar to the results from the marker analysis performed in chemically defined conditions. Differentiated cells staining positive for Mixl and Lim1 in the nucleus could be detected in Activin cultures. Similarly, Hand1 and placental Cadherin (P-cad) positive giant cells could also be derived from SB treated ES cells. Control treatments with a low dose of DMSO carrier (1/5000 dilution) contained large populations of ES cells that stained strongly for Oct4 and SSEA-1. These results confirmed that the level of Nodal/Activin signaling is responsible for at least 3 cell fate decisions. The endogenous level of signaling is permissive for self-renewal and maintenance of pluripotency, an increase in signaling leads to the induction of mesendoderm like cells while reduction of signaling results in trophectoderm differentiation.

### Differential Levels of Nodal/Activin Signaling Regulates the Expression of Distinct Subsets of Target Genes

We hypothesized that for divergent differentiation programs to be initiated in ES cells, differential gene expression mediated by pSmad2 transcription would be a pre-requisite, which is in turn dependent on the level of Nodal/Activin signaling. Each discrete signaling threshold should induce an independent and unique transcriptional signature distinct from other thresholds. To determine the genetic targets regulated downstream of Nodal/Activin signaling and their pattern of expression, microarray analysis was carried out to examine genome-wide gene expression following Activin, DMSO or SB treatments in chemically defined KSR media for 18 hours.

**Figure 3:**
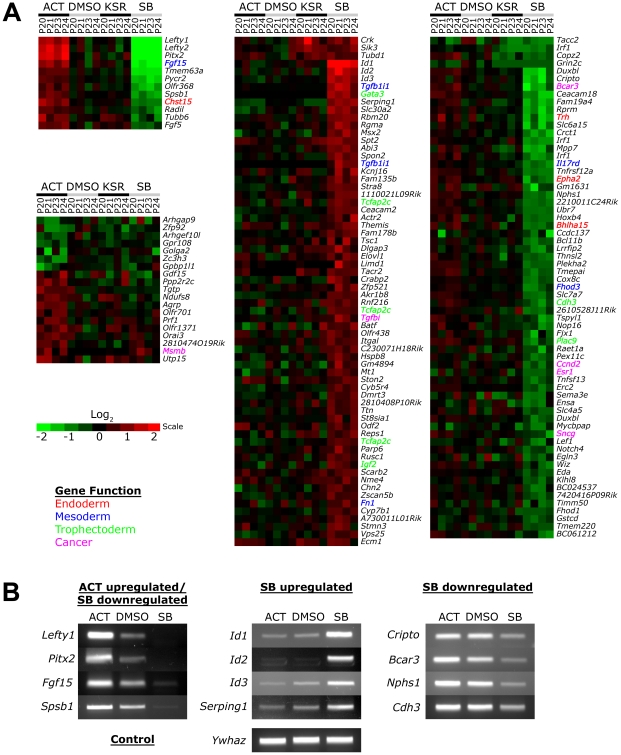
Increased and Decreased Nodal/Activin Signaling Leads to Transcriptional Regulation of Specific Subsets of Target Genes. (A) Heatmap showing microarray analysis of ES cells after 18 hours treatment with Activin (ACT), SB or DMSO vehicle control in KSR media. Transcript levels are expressed as log_2_ fold change over the average of DMSO controls with red showing upregulation and green showing downregulation in n = 4 biological replicates from passage numbers 20 to 24 (P20-P24). Highlighted gene names indicate known roles or domains of expression in endoderm (red), mesoderm (blue), trophectoderm (green) or cancer (magenta). Color bar shows fold change in gene expression on a log_2_ scale. (B) RT-PCR validation of target genes identified in the microarrays that are specifically regulated in ACT and/or SB treatments compared to the DMSO control. *Ywhaz* was used as a housekeeping control.

No significant changes in gene expression out of 26,000 probes could be detected between the DMSO and KSR media control suggesting that the effect of the low concentration of DMSO was negligible on ES cells (Figure 3A). In contrast, Activin and SB treatments induced specific changes in gene expression compared to the DMSO and KSR media controls. Most significantly, we were able to identify subsets of target genes that were regulated by one signaling level and not the other consistent with our hypothesis of threshold specific target gene regulation. For example, 19 genes including *Gdf15*, *Msmb* and *Orai3* were consistently upregulated in Activin treated cells while showing no significant changes in SB. In contrast, a larger subset of 131 target genes were specifically up- and down-regulated only in the SB treatment and not in Activin. A core subset of 12 targets was co-regulated by both high and low signaling changing their expression in correlation with the treatment including the known Nodal/Activin target genes *Lefty1/2* and *Pitx2* that were upregulated by Activin and downregulated in SB.

Interestingly, the number of SB regulated targets significantly exceeds that of Activin targets, suggesting that endogenous Nodal/Activin signaling in ES cells is high or near saturation levels such that a 2-fold increase in pSmad2 could only induce a smaller subset of genes compared to a 2-fold downregulation. Higher doses of Activin treatments and greater than 2-fold increases in pSmad2 may be required to mirror the strength of SB inhibition providing an explanation for asymmetric up- or downregulation of gene expression during different levels of signaling. Some of the target genes driven by Nodal/Activin signaling were indeed implicated in the mesoderm, endoderm and trophectoderm lineages. *Fgf15* plays an important role in the development of cardiac mesoderm [43] and *Chst15* is specifically expressed in definitive endoderm *in vivo* [44] with both targets being upregulated by Activin. For SB treatments, *Gata3*, *Tcfap2c* and *Igf2* were specifically upregulated. *Gata3* is a driver of trophectoderm development [45], [46] while *Tcfap2c* is expressed specifically in the placenta where it regulates essential *ADA* expression [47], [48] and *Igf2* is an imprinted gene that modulates nutrient supply between the placenta and fetus [49], [50]. Together these target genes support some of the mesendodermal and trophectodermal differentiation programs that may be initiated at 18 hours after the induction of differential Nodal/Activin signaling. With longer-term graded Nodal/Activin signaling over 6 days differentiation, it is likely that additional target genes reinforcing the specification of both lineages may be brought into play over time.

*Lefty1*, *Pitx2*, *Fgf15* and *Spsb1* were validated by RT-PCR (Figure 3B) to be co-regulated target genes of high, medium and low signaling displaying a gradient of expression following the signaling level. *Cripto*, *Bcar3*, *Nphs1* and *Cdh3* were targets that were predominantly downregulated by SB inhibition of signaling showing no significant change during Activin stimulation. Conversely, the *ID1*/*2*/*3* family of transcriptional repressors and *Serping1* are specifically upregulated only by the SB treatment showing no difference in response to either Activin or the DMSO control. Hence we conclude that different thresholds of Nodal/Activin signaling are indeed able to regulate the expression of specific subsets of target genes providing an important explanation for the establishment of divergent differentiation programs.

### Phospho-Smad2 Binding to Target Gene Subsets and Regulatory Elements Changes Dynamically Depending on the Level of Nodal/Activin Signaling

**Figure 4:**
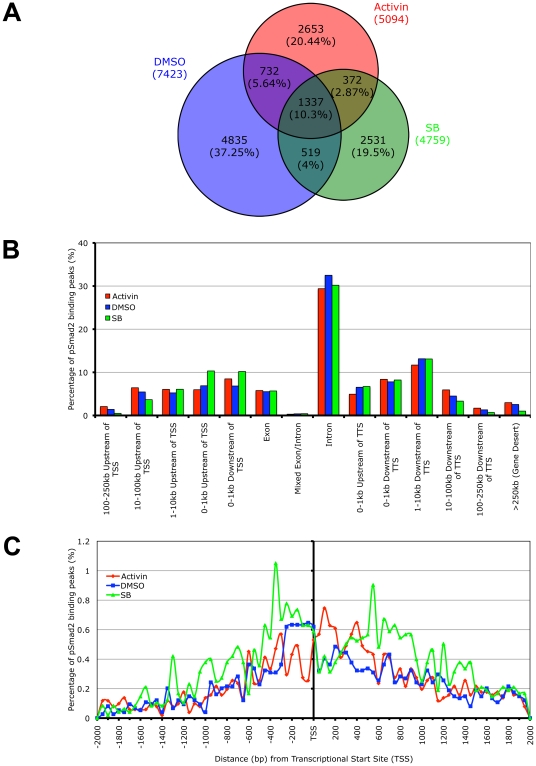
Phospho-Smad2 Binding to the Global ES Cell Genome Changes Dynamically in a Dose-Dependent Manner. (A) Proportional Venn diagram of overlapping and treatment specific pSmad2 binding peaks identified by ChIP-Seq from same passage ES cells cultured for 18 hours in Activin (ACT), DMSO control or SB in KSR media. Overlaps were defined using genomic coordinates of the start and end of peak positions identified with the MACS program using the same parameters for each treatment. Total numbers of peaks are stated for each condition and percentage of peaks in each Venn diagram segment is indicated in parentheses. (B) Graph showing frequency of pSmad2 peaks with respect to distribution in the indicated genomic locations including the transcriptional start sites (TSS) and transcriptional termination sites (TTS) relative to mouse RefSeq genes. Frequency is expressed as percentage of all pSmad2 binding peaks in conditions of high (Activin), medium (DMSO) and low (SB) signaling. (C) High resolution view of pSmad2 binding frequency within +/−2 kb of the TSS of target genes. Genomic distance is expressed in base pairs (bp) upstream (negative) and downstream (positive) of the TSS while frequency is shown as percentage of pSmad2 binding peaks binned in 50 bp intervals out of the total number of peaks for each treatment.

While whole genome microarrays are able to identify the putative subsets of target genes differentially expressed during specific Nodal/Activin signaling levels, this does not provide a molecular mechanism for how different target genes can be directly regulated by the same pathway at different signaling strengths. To address this question, we examined the recruitment of the pSmad2 transcription factor to target genes after subjecting ES cells to Activin, SB or DMSO control treatments in chemically defined KSR media that produce 2-fold up- and downregulation of Smad2 phosphorylation by 18 hours. ChIP-Seq of pSmad2 was employed to identify where pSmad2 was binding on a whole genome scale in parallel cultures of ES cells under the 3 signaling conditions. ChIP samples from each condition were sequenced to a similar depth of 10-13 million tags. Interestingly the number and magnitude of pSmad2 binding events did not correspond to the 2-fold up- or downregulation of pSmad2 in ES cells under Activin and SB treatments. In fact the greatest number of binding peaks (7423) occurred in the control DMSO condition that maintains self-renewal and pluripotency of ES cells (Figure 4A). When homeostatic Nodal/Activin signaling was perturbed by Activin and SB treatments, the number of binding events decreased to 5094 and 4859 respectively suggesting that any change in the levels of endogenous pSmad2 from the ES cell undifferentiated condition also caused a dynamic change in pSmad2 binding across the ES cell genome. The lower numbers may also be reflective of the transition where pSmad2 is dissociating from former target genes and establishing the recruitment of new genes. This was further supported by the percentage of overlapping peaks that were common to the 3 treatments being relatively small at 10.3% with a significantly larger number of unique peaks appearing in specific treatments (37.25% in DMSO, 20.44% in SB and 19.5% in Activin out of 12979 total peaks in the union).

A previous study has profiled Smad2/3 binding sites using promoter arrays in human keratinocytes [31]. However, reporter assays on Nodal/Activin responsive target genes such as *Lefty1/2*, *Nodal* [36], [51] and *Pitx2* [35] suggest that Smad2/3 may also regulate DNA elements in the introns rather than at the promoter region. Consistent with the reporter assay studies, our ChIP-Seq data showed that the majority of pSmad2 binding (Figure 4B) occurs in introns (∼30%) with only a minority of sites in the proximal promoters (∼10%). Furthermore, there was a significant shift in pSmad2 binding from the distal 5′ and 3′ regions towards the promoters of genes in the SB treatment compared to DMSO and Activin (Figure 4B). Examination of binding specifically in the promoter region showed a clear preference for pSmad2 to associate in the +/−600 bp proximal region of transcriptional start sites (TSS) with a steady decrease in binding further away from the TSS (Figure 4C). In addition, the increase in number of pSmad2 binding peaks during low signaling with the SB treatment can be confirmed in the promoter region both up- and downstream of the TSS. In conclusion, pSmad2 binding, similar to the changes in gene expression identified by microarrays, also demonstrates binding to distinct subsets of genomic locations at different signaling levels.

### Phospho-Smad2 Binding Is Positively Associated with Transcriptional Activity and Its Binding Preference for Specific DNA Motifs Changes with Signaling Levels

**Figure 5:**
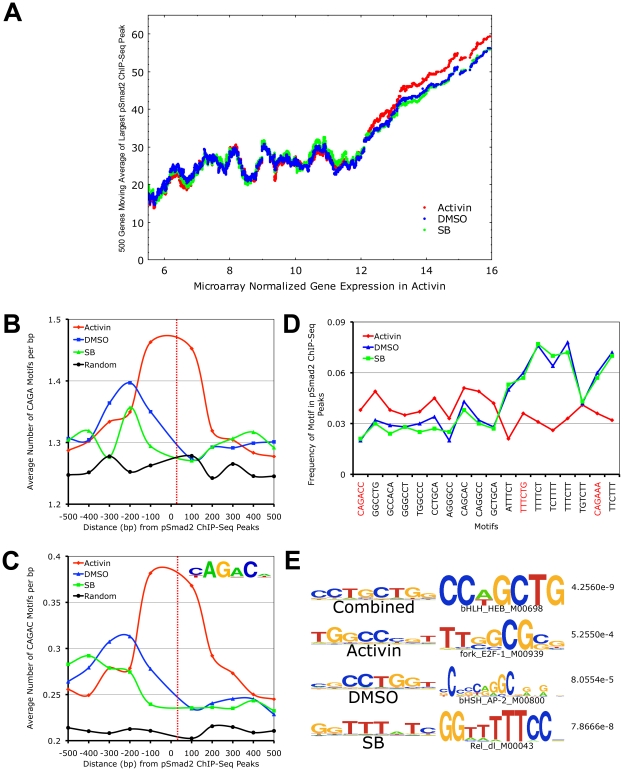
Target Gene Expression Increases with Phospho-Smad2 Binding, Which Demonstrates Dose-Dependent Affinity for Specific DNA Motifs. (A) Plot of relationship between pSmad2 binding affinity and expression levels of associated target genes in the Activin, DMSO and SB conditions. Horizontal axis shows ∼8000 RefSeq microarray targets ranked based on normalized log_2_ expression with a cutoff of 5.5 to exclude non-expressing genes and non-functional probes. The largest pSmad2 ChIP-Seq peaks occurring within +/-50 kb of the RefSeq genes were identified for each condition, normalized to average DMSO enrichments and subjected to a 500 gene moving average calculation on the vertical axis. (B) Plot showing frequency distribution of basic CAGA Smad-binding DNA element (SBE) sequence in 100 base pair (bp) intervals up- and downstream of ChIP-Seq peak positions. Horizontal axis shows distance in bp from the center (broken red line) of ChIP-Seq peaks and vertical axis shows frequency of CAGA motifs in each interval. The frequency of CAGA sequences in 10,000 random mouse genome locations was computed as a control for comparison (black line). (C) Plot of frequency distribution for the canonical Smad CAGAC sequence in intervals around ChIP-Seq peak positions using the same control calculations as in (B). Insert shows the logo of the Smad binding motif (TRANSFAC PWM SMAD_Q6_01) containing the CAGAC sequence. (D) Graph showing the sequence and frequency of the top 6-mer motifs in Activin, DMSO and SB. The motifs were defined by the Weeder program using sequences that occur only in the center of ChIP-Seq peaks. The frequency is expressed as fraction of +/–25 bp sequences spanning ChIP-Seq peaks containing the indicated motifs. CAGA containing 6-mers are highlighted in red. (E) 8-mer motifs defined by Weeder program in top 1000 ChIP-Seq peaks ranked by enrichments in +/−50 bp intervals for Activin, DMSO, SB and combined conditions correspondingly. Transcription factors binding to sequences similar to these motifs identified by the STAMP program using TRANSFAC PWM are indicated with corresponding p-values.

We next examined the relationship governing the degree of pSmad2 binding and the level of transcription across the genome (Figure 5A). In all 3 conditions, a clear trend emerges suggesting that more pSmad2 binding drives higher levels of gene expression. However, the possibility that pSmad2 is not driving expression but preferentially associates with more transcriptionally active genes and open chromatin cannot be excluded. To distinguish between the 2 possibilities, we examined the trend between pSmad2 binding events and differential gene expression from the microarray analysis in the 3 signaling conditions. Indeed, a significant majority (64.2%) of microarray target genes had pSmad2 binding within +/−50 kb and all displayed >1.5 fold change in binding in each signaling condition or had different number of binding events or changed the location of pSmad2 binding (Table S1) suggesting that the pattern of gene expression was indeed dynamically driven by pSmad2-DNA interactions.

To account for how pSmad2 is able to switch binding locations during differential Nodal/Activin signaling, we examined its preference for specific DNA motifs under each condition. It is known that Smad2/3 are able to bind directly the basic CAGA motif and at the same time they possess a number of partner transcription factors that modulate the specificity and strength of binding. Here we see that there is strong pSmad2 association with the basic CAGA SBE specifically in the Activin treatment (Figure 5B). This was also confirmed when we examined the strong CAGAC canonical SBE as defined by the TRANSFAC PWM database which also appears with high frequency at the center of pSmad2 ChIP-seq peaks in the Activin treatment and not in DMSO, SB or the random mouse genome sequence control (Figure 5C). This suggests that both CAGA and CAGAC displayed graded pSmad2 binding that varied with the signaling level and were preferentially bound in the Activin condition. To compare the contribution of CAGA against non-CAGA sequences towards pSmad2 binding, the top 10 *de novo* motifs in each condition were identified using the Weeder program (Figure 5D). Motifs that occurred with significant frequency but were not enriched in the center of pSmad2 ChIP-Seq peaks were excluded to remove the influence of comotifs around the peaks. A number of non-CAGA motifs that occurred with similar or greater frequency than CAGA were isolated. Interestingly, these *de novo* motifs also showed a graded effect on pSmad2 association similar to the CAGA SBE. Other non-CAGA motifs were preferentially bound by pSmad2 only in the DMSO and SB condition and depleted during the high signaling Activin condition. This suggested that while CAGA binding was significant, binding to non-CAGA sequences accounted for the majority of pSmad2 association within the ES cell genome suggesting that this was primarily mediated by transcriptional co-partners. Indeed, when the top consensus motifs in the center of all ChIP-seq peaks in each signaling condition and in the combined dataset were studied (Figure 5E), there was a strong enrichment for motifs belonging to transcription partners such as E2f and Ap1 instead of Smad binding CAGA boxes. To confirm the association of the putative transcriptional cofactors and establish their identity, we expanded the analysis to TRANSFAC co-motifs occurring within +/− 1 kb range of pSmad2 binding sites (Table S2). A large number of known pSmad2 transcription partners such as Ap1, Sp1 and E2f are indeed associated within the vicinity of pSmad2 peaks regardless of the level of Nodal/Activin signaling. However, there were additional co-motifs bound by transcription factors such as Oct4, Stat3 and p53 that only appear prominently in Activin treatments and Hes1, Lrf and Plzf appearing in SB. This is supportive of an exchange of transcription partners in association with pSmad2 that was governed by the level of Nodal/Activin signaling which was likely to be responsible for the change in specificity of pSmad2 transcriptional complexes for target gene subsets and their level of expression. Furthermore, while pSmad2 does bind to its own CAGA sequence, transcriptional copartners played a greater role both in binding affinity and specificity of pSmad2 protein complexes for the ES cell genome.

### Graded, Low Signaling Dominant, High Signaling Dominant, and Multimodal Models of Binding Occur for Phospho-Smad2 Target Genes

**Figure 6:**
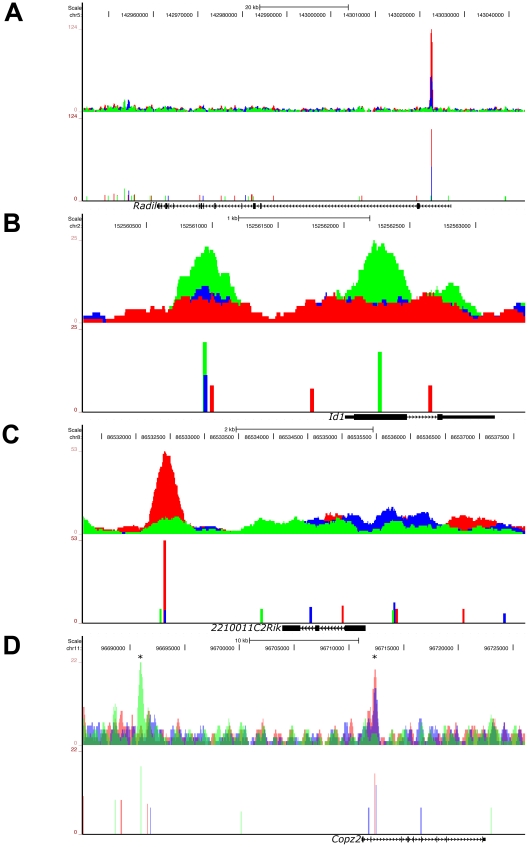
Graded, Low Signaling Dominant, High Signaling Dominant, and Multimodal Modes of Phospho-Smad2 Target Gene Binding. UCSC Genome Browser representation of pSmad2 enrichment peaks under Activin (red), DMSO control (blue) and SB (green) signaling conditions in ES cells. Genomic locations of signaling regulated peaks correspond to (A) graded binding to intron of *Radil* in all 3 conditions, (B) discrete binding to promoter and exon of *Id1* specific to SB treatment, (C) binding to the 3′ region of *2210011C2Rik* only in the Activin condition and (D) multimodal recruitment of pSmad2 in ChIP-Seq peaks marked with (*) where it is SB specific in the promoter region of *Copz2* and graded in correlation with signaling in the intronic region. All target genes were identified to be differentially expressed under the 3 signaling conditions in the microarray analysis of Figure 3A. Top panels show raw sequencing tag enrichments in Activin/DMSO/SB on the vertical axis. Bottom panels show ChIP-Seq peak positions and intensities defined by the MACS program based on normalization to the respective input DNA sequencing controls for each condition. Horizontal scale shows genomic coordinates on the indicated chromosomes and scale bar denotes genomic distance. Structure of the indicated target genes is represented by thick solid lines for exons, thin solid lines for UTRs and continuous arrows running from 5′ to 3′ for introns.

To investigate the different models of pSmad2 binding during differential Nodal/Activin signaling, we examined the ChIP-Seq profiles including those of the transcriptionally regulated microarray targets and identified at least 4 types of pSmad2 binding. The first model is that of “graded” target genes that follow closely the changes in Nodal/Activin signaling with increased binding and transcription during high signaling, have moderate response in endogenous baseline signaling and showed a loss of binding with decreased mRNA levels during signaling repression. This category of pSmad2 binding comprises 23.87% of high confidence ChIP-Seq peaks corresponding to 16.28% of target genes associated within +/−50 kb of these peaks (Figure S4). *Radil* a Rap GTPase effector that plays a role in the migration of neural crest progenitors [52] exemplified such pSmad2 binding and transcriptional regulation (Figure 3A, Figure 6A, and Table S1) in the first intron with normalized relative enrichments of 107 tags in Activin compared to 51 in the DMSO control and complete loss of binding indistinguishable from background sequencing levels in SB. The known target gene *Pitx2* showed reproducible results with the ChIP data obtained by real-time PCR (Figure S1A) both in terms of the binding location in the intronic enhancer as well as the level of pSmad2 enrichments under graded Nodal/Activin signaling. There were normalized enrichments of up to 201 tags in Activin, 156 in DMSO control and again complete loss of binding in SB (Figure S3A). Interestingly, *Pitx2* had 2 graded binding sites, one of which is in the known intronic region and a novel site in the 3′ region. The graded binding in the *Pitx2* locus also correlates with transcriptional consequences showing strong induction/inhibition of *Pitx2* mRNA levels from 0 to 24 hours (Figure 1A).

The two inducers of the mesendoderm cell fate *Mixl* [53] and *Nodal* [54] also show evidence of graded pSmad2 binding within 50 kb of the genes (Figure S5A and S5B) suggesting that they may be directly regulated by Nodal/Activin signaling for this purpose. The binding location in the first intron of *Nodal* also corresponds to the intronic enhancer previously described to be important for left side expression in the early embryo via the Nodal/Activin signaling autoregulatory loop [55], [56] confirming that *Nodal* is itself a direct target.

It was also unclear if pSmad2 binding and regulation of target genes only exists in a 1-to-1 relationship or if the same binding sites were capable of regulating multiple targets in the genomic vicinity. While *Lefty2* was a known direct target with pSmad2 binding in its promoter region (Figure S1B), for the first time, to our knowledge, we characterized an important pSmad2 transcriptional hotspot in the entire 100 kb *Lefty1/2* locus where all the genes within this region were co-regulated by pSmad2 binding suggesting a coordinated mode of transcriptional regulation (Figure S3B). This was further confirmed in the microarray analysis (Figure 3A) demonstrating that *Lefty1/*2, *Pycr2* and *Tmem63a* display the same pattern of gene expression following a graded response to Nodal/Activin signaling. This was consistent with the real-time PCR quantification of the pSmad2 pulldown of the *Lefty2* promoter (Figure S1B) that corresponds to the most upstream pSmad2 binding site in the *Lefty1/2* hotspot as did a time course profiling of *Lefty2* expression from 0 to 24 hours (Figure 1B).

In the second model of pSmad2 binding, we describe “low signaling dominant” conditions that permit pSmad2 binding but less so under other signaling levels. The *Id1/2/3* (Figure 6B, Figure S6, and Table S1) family of transcriptional repressors shows pSmad2 binding to these genes only in the SB treatments and not in Activin or the DMSO control. Statistically, 32.73% of pSmad2 binding sites display this mode of behavior associated with 23.44% of target genes (Figure S4). In contrast, the third model showed the opposite “high signaling dominant” mode of binding such as in the case of *220011C2Rik* (Figure 6C and Table S1) where pSmad2 only binds strongly in the Activin condition but to a lesser degree in DMSO or SB also resulting in transcriptional consequences (Figure 3A and Figure S4). Another known component of the mesendodermal cell fate *Fgf8* [57] also shows strong pSmad2 binding in the promoter region specifically during high signaling (Figure S5C). Intriguingly, findings in the chick embryo show that *Fgf8* also plays important roles in left-right asymmetry where it can be induced by Activin [58] in agreement with our results.

In the fourth model which accounts for the regulation of the largest proportion (33.69%) of target genes associated with pSmad2 ChIP-Seq peaks (), the same target gene may be regulated by “multimodal pSmad2 binding” events. *Copz2* has two pSmad2 association sites in the intron and promoter region (Figure 6D). The promoter site only binds pSmad2 in the SB condition while the intronic enhancer shows a graded response to the signaling level. In the case of the known target gene *Smad7*, we have shown that the pSmad2 binding peak in the promoter region is invariant in all 3 signaling conditions (Figure S1C) which could not explain how *Smad7* was differentially expressed during graded Nodal/Activin signaling (Figure 1C). In confirmation with these results, the ChIP-Seq data showed the same pSmad2 association on the *Smad7* proximal region with no change in binding under all 3 signaling conditions. Surprisingly, we discovered a previously undescribed pSmad2 regulatory element in the distal *Smad7* promoter region that binds pSmad2 in a graded manner (Figure S3C) and could account for why *Smad7* was responsive to different Nodal/Activin signaling levels. Hence pSmad2 binding in the *Smad7* proximal region may not be the dominant regulatory region for Nodal/Activin signaling but may depend instead on the dynamically changing pSmad2 distal promoter element for *Smad7* regulation. Indeed the proximal promoter element may be more of a Smad3 regulated region instead of Smad2 as previously described [38].

In conclusion we demonstrate that pSmad2 dependent binding and transcription during graded Nodal/Activin signaling occurs in the ES cell genome in a graded, low or high signaling dominant, many-to-one or one-to-many multimodal manner in relation to the target genes that they regulate.

### Nodal/Activin Signaling Titrates the Level of *Oct4* in ES Cells, Which Is a Direct Target of Phospho-Smad2 Binding in the Promoter Region

The mesendodermal and trophectodermal cell fate decisions brought about by graded Nodal/Activin signaling strikingly resemble the ES cell response to a less than 2-fold up- or downregulation of the *Oct4* master regulator of stemness in driving differentiation towards similar cell fates [59]. Furthermore, an important mechanism for trophectoderm differentiation depends on the Oct4 repression of *Cdx2* expression and the induction of this lineage is thought to be indicative of loss of stemness [60]. We therefore hypothesized that *Oct4* may be a key downstream target under Nodal/Activin control during the specification of divergent cell fate decisions and investigated how the pathway may be governing *Oct4*.

**Figure 7:**
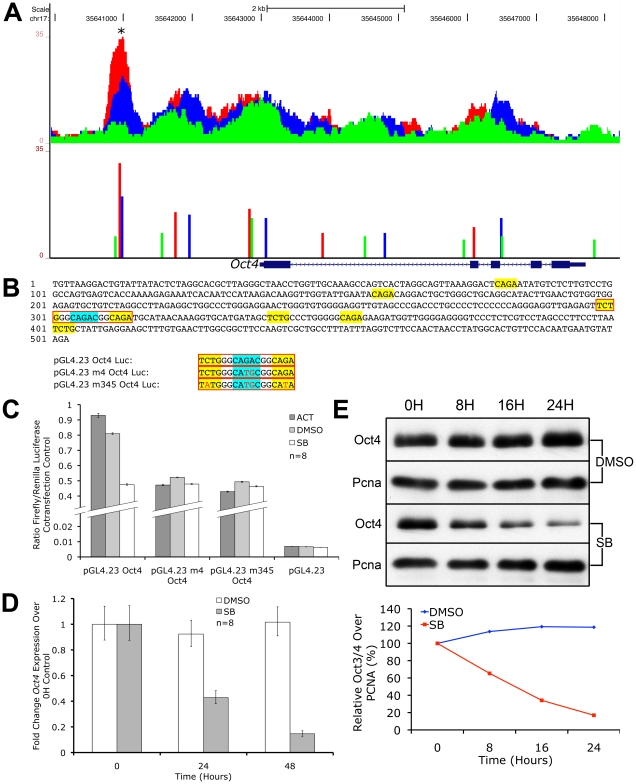
*Oct4* Is a Direct Target of Nodal/Activin Signaling via Graded Phospho-Smad2 Recruitment to the Promoter. UCSC Genome Browser plot of the *Oct4* genetic locus showing pSmad2 ChIP-Seq enrichment on the vertical scale in ES cells subjected to Activin (red), DMSO control (blue) and SB (green) treatments with genomic distance in bp on the horizontal scale. Colored bars show ChIP-Seq peak positions and normalized enrichments for each treatment. (B) 503 bp sequence of the *Oct4* promoter region marked with asterisk (*) in (A). Sequences in yellow show canonical CAGA SBEs or their inverted sequence TCTG while the strong CAGAC SBE is highlighted in blue. Sequences in red boxes denote where mutations were made in the indicated luciferase constructs. (C) Firefly luciferase assays of the 503 bp sequence cloned into the *pGL4.23* reporter construct (*pGL4.23 Oct4*) or mutated in the strong CAGAC SBE (*pGL4.23 m4 Oct4*) or mutated in CAGAC and the two flanking CAGA sites (*pGL4.23 m345 Oct4*). The constructs were transfected into ES cells treated with Activin (dark gray bars), DMSO control (light gray) and SB (white). Discontinuous vertical scale shows relative Firefly luciferase levels normalized to the co-transfection *pGL4.75* Renilla luciferase control. Error bars provide s.e.m. for n = 8 replicates. (D) Real-time PCR quantitation of endogenous *Oct4* expression in ES cells treated for 0 to 48 hours in SB (gray bars) compared to DMSO (white bars) after normalizing to the *Ywhaz* housekeeping control. Vertical axis shows fold change over the 0 h control in each treatment. Error bars show s.e.m. for n = 8 replicates. (E) Western blot of endogenous Oct4 protein levels in ES cells after treatment with SB or DMSO control in a 0 to 24 hour time course. Pcna was used as a loading control in both conditions. Densitometry plot shows Oct4 protein quantitation after normalizing to the respective Pcna loading control with the relative level at 0 hours for each treatment at 100%.

We discovered that the *Oct4* locus was rich in multiple pSmad2 binding events from ChIP-Seq profiling (Figure 7A). During graded Nodal/Activin signaling in chemically defined conditions however, only a pSmad2 peak in the promoter region of *Oct4* showed a similarly graded response suggesting that this was the functional Nodal/Activin signaling response element. We examined the transcript levels of endogenous *Oct4* expression (Figure 7D) upon inhibition of Nodal/Activin signaling with SB in serum containing media and found that it was also significantly downregulated within 24 hours. In agreement with the transcript data, Oct4 protein levels were similarly downregulated in SB treated ES cells (7E). Analysis of the 503 bp promoter region encompassing the beginning and end of the pSmad2 binding peak showed that it contained eight CAGA sites or their inversion (Figure 7B). To determine if this regulatory sequence was indeed a Nodal/Activin response element of the *Oct4* promoter, we cloned this into luciferase reporter constructs and transfected ES cells subjected to the 3 signaling conditions with Activin, DMSO and SB (Figure 7C) in serum containing media. The reporter activity of the wild type *Oct4* promoter construct was >100X higher than that of the empty reporter construct in the DMSO control signaling condition suggesting that the 503 bp sequence had strongly driven *Oct4* promoter activity in ES cells. Crucially, the *Oct4* promoter reporter displayed a specific graded response to Nodal/Activin signaling while the control empty reporter did not. To confirm that the *Oct4* response to graded Nodal/Activin signaling was functionally driven by pSmad2 binding, we determined the exact SBE responsible for *Oct4* inducibility (Figure 7C). Mutagenesis experiments on the *Oct4* promoter region in luciferase assays revealed that the strong CAGAC consensus SBE site in the middle of the 503 bp fragment was indispensable for graded *Oct4* promoter activity. Loss of this site completely abolished the promoter response to both high and low Nodal/Activin signaling. Further point mutations of two minimal CAGA SBEs flanking the CAGAC site led to no further significant effects on the *Oct4* promoter.

We therefore conclude that *Oct4* is a direct target of pSmad2 binding and Nodal/Activin signaling regulates both its promoter activity and endogenous expression. The 503 bp *Oct4* promoter response element with the essential CAGAC SBE was sufficient and independent of all other pSmad2 binding events in the *Oct4* locus or other DNA regulatory elements in cis that may be mediated by Nodal/Activin signaling. The regulation of *Oct4* is well known for its importance in cell fate decisions and its downregulation during loss of Nodal/Activin signaling is significant not only as an impetus for trophectoderm differentiation but also reconciles the alternative role of Nodal/Activin signaling in maintaining self-renewal and pluripotency.

## Discussion

The molecular basis of extracellular signaling instructions governing differential cell fate decisions in the Nodal/Activin pathway has been postulated but not shown conclusively. Primarily, the transcriptional events occurring at the interface between pSmad2 signal transduction from the activated cell surface receptors to manipulation of the global stem cell transcriptome driving specific lineage programs have not been well characterised. This study provides an important insight into how quantitative signaling is translated into qualitative cell fate decisions by showing for the first time, to our knowledge, that the same transcription factor pSmad2 is able to bind and transcriptionally regulate different subsets of target genes in a dose-dependent manner.

The specification of cell fate decisions is governed by 2 distinct events. The first requires an exit from self-renewal and maintenance of stemness programs by direct control of pSmad2 over key pluripotency factors. Previous studies have revealed that *Nanog* is a direct target of Smad2/3 transcription in human ES cells [61]. Here we show an additional level of control over the stem cell program by direct transcriptional regulation of the *Oct4* master pluripotency gene by pSmad2. The second event requires an entry into a specific differentiation program that is in turn brought about by direct and indirect pSmad2 regulation of differentiation genes such as *Gata3*, *Tcfap2c* and *Igf2* that are known to be important factors for trophectoderm cell fates. This cell fate decision is further reinforced by loss of *Oct4* with inhibition of Smad2 phosphorylation as the former is known to be a potent repressor of the trophectoderm gene *Cdx2* in the blastocyst [60]. The pSmad2 binding target genes driving mesendodermal differentiation include *Mixl*, *Fgf8* and *Nodal* itself, while other genes such as *Chst15* expressed in definitive endoderm and *Fgf15* for cardiac mesoderm have also been identified as strong Nodal/Activin transcriptional targets. It is likely that over the course of long-term differentiation for 6 days, additional target genes may be recruited for the specification of both lineage decisions that may not be apparent at the 18 hours time point in this study which may be too early for endpoint differentiation. Indeed, strong regulation of *Mixl* and *Fgf8* and to a lesser extent *Nodal* could be detected at 3 and 6 days (Figure 2B and data not shown) of treatment in correlation with the level of Nodal/Activin signaling.

**Figure 8:**
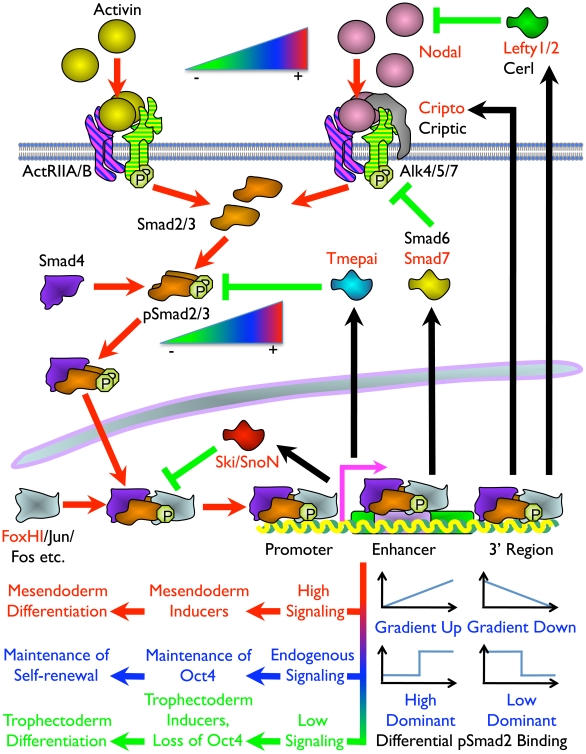
Model of the Mechanism of ES Cell Fate Decisions Directed by Graded Nodal/Activin Signaling. Schema of the components and signal transduction of the Nodal/Activin pathway starting with different concentrations of ligands external to the ES cell lipid bilayer membrane and terminating with the pSmad2 transcriptional complex regulating *Oct4* and different subsets of target genes in the nucleus. Red arrows show the signal transduction circuit, black arrows show transcription and translation of pSmad2 target genes while green inhibitory lines indicate negative feedback. Protein names in red are targets identified in the microarray analysis and/or pSmad2 ChIP-Seq. Color gradients from red to green denote components exhibiting a dose-dependent response from high (+) to low (–) activity. Plots represent the graded, high and low signaling dominant models of pSmad2 binding during differential signaling with the vertical axis showing the level of binding against the horizontal axis with increasing signaling levels from left to right. Different cell fate decisions and the events triggering them are indicated by lines and arrows in red to green color gradients.

Consistent with the role of Nodal/Activin as morphogens, we found that many components of the pathway were themselves feedback targets that were directly regulated by pSmad2 binding in ChIP-Seq and/or differentially expressed in our microarray analysis. These include the negative feedback inhibitors such as *Lefty1/2* and *Smad7* which are already known targets of Nodal/Activin signaling. In this study, graded pSmad2 binding could be detected in the intronic region of *Tmepai* (Figure S6) which sequesters Smad2/3/4 from receptor kinase activity [62]. Similarly, *SnoN* [63] and *Ski* [64] also present graded intronic binding of pSmad2 (Figure S6) and both function as transcriptional repressors of Smad2/3/4. There were also positive feedback components such as *Nodal*, its cofactor *Cripto* and *FoxH1* the transcriptional copartner of Smad2 (Figure 8 and Figure S6) that show graded binding in the intron and promoter regions. The preponderance of the negative components in the autoregulatory loop of Nodal/Activin signaling is significant, as it suggests that the pathway mainly dampens and attenuates its own signaling via negative feedback and less so by positive feedback loops mediated by *Nodal, Cripto* and *FoxH1.*

One of the intriguing findings is that extracellular signaling gradients were translated into a gradient of Smad2 phosphorylation that we have now shown to be able to recruit different target genes in a dose-dependent manner. This was possibly achieved by an exchange of transcriptional copartners that permits the shifting of the pSmad2 transcriptional complex to different target gene subsets as suggested by the differential recruitment of non-CAGA motifs and comotifs under each signaling condition. The fact that pSmad2 contains only CAGA sequence binding domains and not transcription activation domains suggest that it is further dependent upon copartners for transcription, binding affinity and specificity. In some cases graded pSmad2 transcription complex binding drives graded target gene response that follows signaling strength with high fidelity. In other cases, the target genes are only regulated and responsive at defined signaling thresholds (Figure 8). The consequence is that a relatively modest stimulation with Activin leading to a physiological 2-fold increase in Smad2 phosphorylation eventually drives mesendodermal differentiation while the reciprocal SB inhibition resulting in a 2-fold decrease of pSmad2 is able to promote trophectoderm cell fates. During this process, the master regulator of pluripotency *Oct4* is itself titrated by the same Nodal/Activin signaling gradients in the ES cells undergoing differentiation. Hence the same pathway is able to tilt the balance in favor of maintenance of pluripotency or mediate an exit from self-renewal and entry into a specific lineage program. In conclusion, this study for the first time, to our knowledge, reconciles the multiple divergent roles of Nodal/Activin signaling in both pluripotency and differentiation with pSmad2 playing a central role in the cell fate decision making process.

## Materials and Methods

### Cell Culture

E14 TG2A mouse embryonic stem cells (ATCC) were propagated in FBS media consisting of 20% ES cell-qualified FBS in DMEM supplemented with 100 µM non-essential amino acids, 100 U/ml penicillin, 100 µg/ml streptomycin, 2 mM GlutaMAX-I (Invitrogen), 55 µM β-mercaptoethanol (Sigma) and 1X homemade Leukemia inhibitory factor (LIF). For the establishment of Nodal/Activin signaling gradients in chemically defined conditions, KSR media containing 20% Knockout Serum Replacement (KSR, Invitrogen) in place of FBS with all other components of ES media excluding LIF were used. For acute (0 to 48 hours) signaling conditions, 25000 ES cells/cm^2^ were plated for 18 hours in FBS media followed by adaptation of the cells to chemically defined conditions with 10 µM SB-431542 (Tocris) in KSR media for 6 hours as previously described [41]. High signaling was induced by treatment with KSR media containing 25 ng/ml Activin (R&D Systems) or low signaling with 10 µM SB and maintenance of endogenous signaling with control KSR media or 1/5000 dilution of DMSO vehicle as indicated. For long-term differentiation, 2000 ES cells/cm^2^ were plated and 18 hours later directly treated with Activin, DMSO and SB in FBS media without LIF or KSR media for 6 days with media change everyday. The DMSO vehicle used to dissolve SB can induce differentiation and loss of pluripotency in ES cells [65], [66]. In the microarray analysis of the 3 signaling conditions, the effect of DMSO on differential gene expression was determined by comparing against the unsupplemented KSR media control (Figure 3A). The SB inhibitor was used at a high stock concentration of 50 mM permitting 5000X dilution of DMSO in ES cell cultures which was well below the limit required for differentiation. The cultures and treatments were carried out for the microarray study in 4 biological replicates consisting of ES cells at 4 different passages from P20 to P24 to identify and eliminate any cell culture variation effects from analysis.

### Western Blotting

ES cells were lysed in RIPA buffer (150 mM NaCl, 1% NP-40, 0.5% Sodium Deoxycholate, 0.1% SDS, 50 mM Tris pH 8.0) for protein extracts. SDS-PAGE was performed on 10% polyacrylamide gels and transferred on Immun-Blot PVDF membranes (Bio-rad Laboratories) followed by probing with 1∶1000 dilutions of rabbit anti-Smad2 (pSer^465/467^, Calbiochem), rabbit anti-Smad2 (Invitrogen), mouse anti-Pcna (Santa Cruz Biotechnology) and goat anti-Oct4 (Santa Cruz Biotechnology). Secondary antibodies used were 1∶1000 donkey anti-rabbit IgG-HRP (GE Healthcare), 1∶1000 goat anti-mouse IgG-HRP and 1∶2500 donkey anti-goat IgG-HRP (Santa Cruz Biotechnology). Densitometry measurements of protein bands on western blots were acquired using Photoshop CS3 (Adobe Systems Incorporated).

### PCR Quantitation of Gene Expression and ChIP DNA Enrichments

For gene expression, total RNA was extracted from cells using the RNeasy Mini kit (Qiagen) as per manufacturer instructions. This was reverse transcribed into cDNA using the High Capacity RNA-to-cDNA Master Mix (Applied Biosystems). Quantitative real-time PCR was performed on the 7900HT Fast Real-Time PCR System (Applied Biosystems) or the Biomark System (Fluidigm Corporation) on cDNA or ChIP DNA according to manufacturer instructions. For RT-PCR, products amplified for 25 to 33 cycles were resolved on a 2.5% agarose gel. Primer sequences for both ChIP-qPCR and gene/marker expression can be found in Table S3.

### Microarray Analysis

Total RNA was reverse transcribed into cDNA and *in vitro* transcribed into biotin-labeled cRNA using the Illumina TotalPrep RNA Amplification kit (Ambion). This was hybridized on MouseRef-8 v2.0 Expression BeadChips (Illumina). Raw intensity values were subjected to background subtraction on the BeadStudio Data Analysis Software (Illumina) and normalized using the cross-correlation method [67]. Differential gene expression was identified based on a fold change cutoff of >1.5 compared to the DMSO control. The microarray data was deposited in NCBI GEO with accession number GSE23239.

### ChIP-Seq

Chromatin Immunoprecipitation (ChIP) using a certified ChIP-grade rabbit polyclonal anti-Smad2 (phospho S465+S467) antibody (ab16509, Abcam) was carried out in ES cells under chemically defined high, medium or low Nodal/Activin signaling conditions according to the Agilent Mammalian ChIP-on-chip protocol v9.1 up to the ChIP DNA purification step. Adapter ligation, library amplification and size selection in the 200–300 bp range were performed according to the Illumina ChIP Sample Prep protocol (#11257047, Rev. A). Massively parallel sequencing was carried out for ChIP samples in all 3 signaling conditions with their respective input DNA controls on the Genome Analyzer (Illumina) up to a sequencing depth of at least 10×10^6^ tags pass filter. The ChIP-Seq data was deposited in NCBI GEO with accession number GSE23581. Details of the ChIP-Seq, motif and statistical analysis can be found in the Text S1.

### Luciferase Assays

The 503 bp fragment of the mouse *Oct4* promoter region corresponding to chr17:35640683–35641185 was cloned into the pGL4.23[luc2/minP] Firefly luciferase reporter construct (Promega) to generate *pGL4.23 Oct4*. This construct was point mutated by oligo cloning into unique StuI and NsiI sites to produce *pGL4.23 m4 Oct4* (CAGAC mutated to CATGC) and *pGL4.23 m345 Oct4* (TCTGGGCAGACGGCAGA mutated to TATGGGCATGCGGCATA). The constructs were transfected into mouse ES cells in an 80∶1 ratio with the pGL4.75[hRluc/CMV] Renilla luciferase co-transfection control (Promega) using Lipofectamine 2000 (Invitrogen). A control transfection was included with an 80∶1 ratio of the empty pGL4.23 vector to pGL4.75 Renilla control. Immediately after lipofections, the ES cells were pretreated with FBS media without LIF and with 10 µM SB for 6 hours. Subsequently the cells were split into replicates and plated in FBS media without LIF containing 25 ng/ml Activin, 1/5000 DMSO vehicle or 10 µM SB, which induces high, medium or low Nodal/Activin signaling respectively for 8 hours. The cells were washed once in PBS and lysed in 1XPassive Lysis Buffer and luciferase assays were performed using the Dual-Luciferase Reporter Assay System on the GloMax 96 Microplate Luminometer with Dual Injectors (Promega).

## Supporting Information

Figure S1 There Is Graded pSmad2 Binding to *Pitx2* and *Lefty2* but Not the *Smad7* Proximal Promoter. Real-time PCR quantification of pSmad2 ChIP enrichments using tiling primers across the intronic enhancer in *Pitx2* (A) and the promoter regions of *Lefty2* (B) and *Smad7* (C). Y-axis shows fold enrichment over the non-binding control region in the *Sox2* locus after normalizing to the input DNA for each condition while the x-axis represents genomic distance in base pairs (bp) from the transcriptional start site (TSS) of each gene. Upstream and downstream distances are denoted as negative and positive coordinates respectively. Trends show the level of pSmad2 ChIP enrichment for the indicated genomic regions obtained from ES cells treated with Activin (red), DMSO carrier control (blue) and SB (red) in chemically defined KSR media. Horizontal bars show coverage of each PCR primer, vertical error bars show s.e.m. for n = 3 replicates.
(TIF)

Figure S2 Activin/SB Treatments of ES Cells Promote Exit from Self-Renewal towards Mesendoderm and Trophectoderm Differentiation. Immunofluorescence detection of the markers of pluripotency and mesendoderm or trophectoderm differentiation in ES cells treated for 6 days with Activin, DMSO control and SB. Phase contrast bright field (BF) images are in the top panels followed by Hoechst 33342 nuclear staining in blue. Alexa Fluor 546 staining for Lim1, Oct4 and Hand1 are in red for Activin, DMSO and SB treated cells respectively. Alexa Fluor 488 staining for Mixl, SSEA-1 and placental Cadherin (P-cad) are in green while overlays of all fluorescent channels (Merge) are shown in the bottom panels. Scale bar shows distance under a 20X objective lens.
(TIF)

Figure S3 *Pitx2*, the *Lefty1/2* Hotspot, and *Smad7* Contain Multiple pSmad2 Regulatory Sites in Their Genetic Loci. UCSC Genome Browser representation of the genomic loci for *Pitx2* (A) on chromosome 3:128,894,461–128,931,307, *Lefty1/2*, *Pycr2* and *Tmem63a* (B) on chromosome 1:182,813,583–182,908,336 and *Smad7* (C) on chromosome 18:75,513,703–75,561,010. Genomic coordinates are shown on the x-axis while raw pSmad2 ChIP-Seq tag counts are presented on the y-axis in the top panel followed by relative enrichments normalized to the respective input DNA controls of Activin (red), DMSO (blue) and SB (green) treated ES cells in the bottom panel. The RefSeq genetic structures of the indicated genes and their isoforms if any are indicated below the ChIP-Seq panels. Scale bar shows genomic distance in kilo base pairs (kb) while pSmad2 enrichment peaks corresponding to the real-time PCR results in Figure S1 are indicated with asterisks (*).
(TIF)

Figure S4 Phospho-Smad2 Binding to Majority of Sites and Target Genes Varies by >1.25-Fold during Graded Signaling. Pie charts showing patterns and statistics of pSmad2 binding to sites and target genes that vary by at least 1.25-fold during Activin, DMSO and SB treatments. Colored segments display the indicated models of pSmad2 binding in the 3 signaling conditions. Proportional segment sizes define the relative contribution of each type of binding behavior for ChIP-Seq peaks (A) and target genes within +/−50 kb of the peaks (B) out of the total for each. Values indicate the number of peaks (A) or genes (B) in each regulatory model with percentages of the total in parentheses.
(TIF)

Figure S5 The Mesendodermal Genes *Mixl*, *Nodal*, and *Fgf8* Are Targets of Differential pSmad2 Binding. Binding profile of pSmad2 ChIP-Seq enrichments on *Mixl* (A), *Nodal* (B) and *Fgf8* (C) as visualized on the UCSC Genome Browser at the coordinates chr1:182,571,609–182,632,748, chr10:60,873,730–60,895,009 and chr19–45:805,804–45,822,014 respectively. Changes in the level of pSmad2 ChIP-Seq peak enrichments (raw peaks top panel, normalized peaks bottom panel) are indicated for Activin (red), DMSO (blue) and SB (green) treated ES cells on the y-axis. X-axis shows genomic location and genetic features of the indicated genes are below the Genome Browser panels. PSmad2 binding peaks showing significant differential binding with Nodal/Activin signaling are marked with asterisks (*). Scale bar shows genomic distance in kilo base pairs (kb).
(TIF)

Figure S6 Validation of 15 Genes as Direct Targets of Nodal/Activin Signaling via Differential Phospho-Smad2 Binding. Real-time PCR validation of pSmad2 ChIP-Seq enrichment on original unamplified ChIP DNA from ES cells treated with Activin (ACT), DMSO and SB in KSR media for 18 hours. Indicated genes were selected based on transcriptional regulation under the same conditions in the microarray analysis of Figure 3A and real-time PCR gene expression detection in Figure 7D. Vertical scale shows fold-enrichment over the non-binding control region in the *Pfkm* locus on chromosome 15:97,934,552–97,934,626 after normalization to the input DNA controls in each treatment. Phospho-Smad2 enriched regions detected by the PCR primers are indicated in the genomic coordinates above each graph. Error bars show s.e.m. for n = 12 replicates.
(TIF)

Table S1 64.2% of Microarray Targets Differentially Expressed by >1.5-fold are also Targets of Differential Phospho-Smad2 Binding. Table showing 104 out of the 162 microarray target genes from Figure 3A where expression is induced and/or repressed by >1.5-fold during stimulation or inhibition of Nodal/Activin signaling in ES cells. These targets also exhibit pSmad2 binding at +/−50 kb from the 5′/3′UTRs and within the genes themselves. Only ChIP-Seq peaks that satisfy a size cutoff of >12 sequencing tags in at least 1 condition are considered significantly above background sequencing levels and counted for each gene during Activin (ACT), DMSO vehicle control and SB inhibitor treatments. Gene IDs of the target genes, their chromosomal locations (Chr) and their orientation on the sense (+)/antisense (−) strands are as indicated. Gene names highlighted in yellow are for Activin/SB co-regulated targets, red for Activin specific targets and green for SB responsive genes only. The number of sequencing tags within the largest ChIP-Seq peaks for each gene and under each condition are as shown. For genes that contain multiple pSmad2 binding sites, the largest peaks counted may not occur at the same genomic location. Changes in the level of overall pSmad2 binding during Activin treatment compared to SB (ACT vs SB), Activin compared to DMSO control (ACT vs DMSO) and SB compared to DMSO (SB vs DMSO) are shown as (+) for increased binding and (–) for decreased binding by >1.5-fold. The overall level of pSmad2 binding for each gene is calculated as the sum of all peak heights (enrichment counts) proximal to the gene and under each condition. A value of 0 denotes no significant change in binding at a 1.5-fold cutoff and NA indicates no detectable peaks for statistical analysis.
(DOC)

Table S2 There Are Dynamic Changes in Phospho-Smad2 Binding Motifs During Graded Nodal/Activin Signaling. MotifEnrich program identification of co-motifs enriched within ≤5000 bp of pSmad2 binding peaks. Co-motifs are ranked based on frequency of occurrence from highest to lowest. Matching of co-motifs with associated transcription factors was performed using the TRANSFAC PWM database with p-value thresholds of 1e-05. Transcription factor names in black are present across all 3 signaling conditions with Activin, DMSO and SB treatments. Activin enriched transcription partners binding on the co-motifs in proximity to pSmad2 sites are highlighted in red, DMSO enriched factors in blue and SB in green. The intervals of significant enrichment for each co-motif are indicated in base pairs (bp).
(DOC)

Table S3 Primers Used for Quantification of ChIP DNA and Marker/Gene Expression Levels by Real-Time PCR. List of forward/reverse primer pairs used in SYBR Green real-time PCR reactions for the quantification of ChIP-DNA (ChIP-qPCR) and Marker/Gene expression. For tiling ChIP-qPCR analysis of pSmad2 binding on *Pitx2*, *Lefty2* and *Smad7*, primers for each gene are numbered sequentially according to the 5′–3′ order of their respective amplified regions shown in Figure S1.
(DOC)

Text S1 Supporting Experimental Procedures and Supporting References. Description of methods used for motif identification and statistical analysis of ChIP-Seq data and immunostaining of the protein markers of differentiation in cells with the associated supplementary references.
(DOC)
