# Supplementary figures and images for: Graded Nodal/Activin Signaling Titrates Conversion of Quantitative Phospho-Smad2 Levels into Qualitative Embryonic Stem Cell Fate Decisions
Source: PLoS Genet. 2011 Jun 23;7(6):e1002130. doi: 10.1371/journal.pgen.1002130 (PMC3121749; doi:10.1371/journal.pgen.1002130)

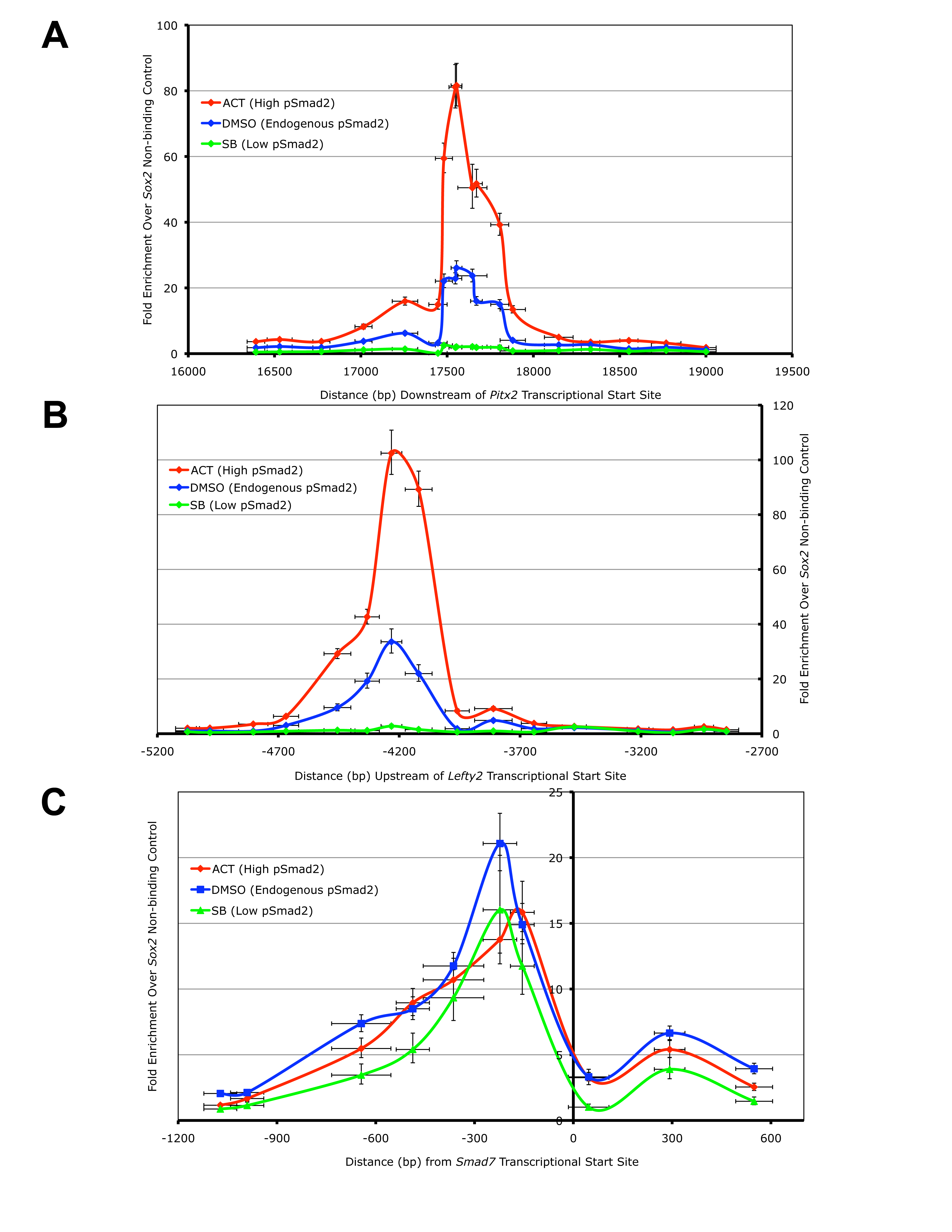

Supplement: Figure S1 — There Is Graded pSmad2 Binding to Pitx2 and Lefty2 but Not the Smad7 Proximal Promoter. Real-time PCR quantification of pSmad2 ChIP enrichments using tiling primers across the intronic enhancer in Pitx2 (A) and the promoter regions of Lefty2 (B) and Smad7 (C). Y-axis shows fold enrichment over the non-binding control region in the Sox2 locus after normalizing to the input DNA for each condition while the x-axis represents genomic distance in base pairs (bp) from the transcriptional start site (TSS) of each gene. Upstream and downstream distances are denoted as negative and positive coordinates respectively. Trends show the level of pSmad2 ChIP enrichment for the indicated genomic regions obtained from ES cells treated with Activin (red), DMSO carrier control (blue) and SB (red) in chemically defined KSR media. Horizontal bars show coverage of each PCR primer, vertical error bars show s.e.m. for n = 3 replicates. (TIF) [file pgen.1002130.s001.tif]

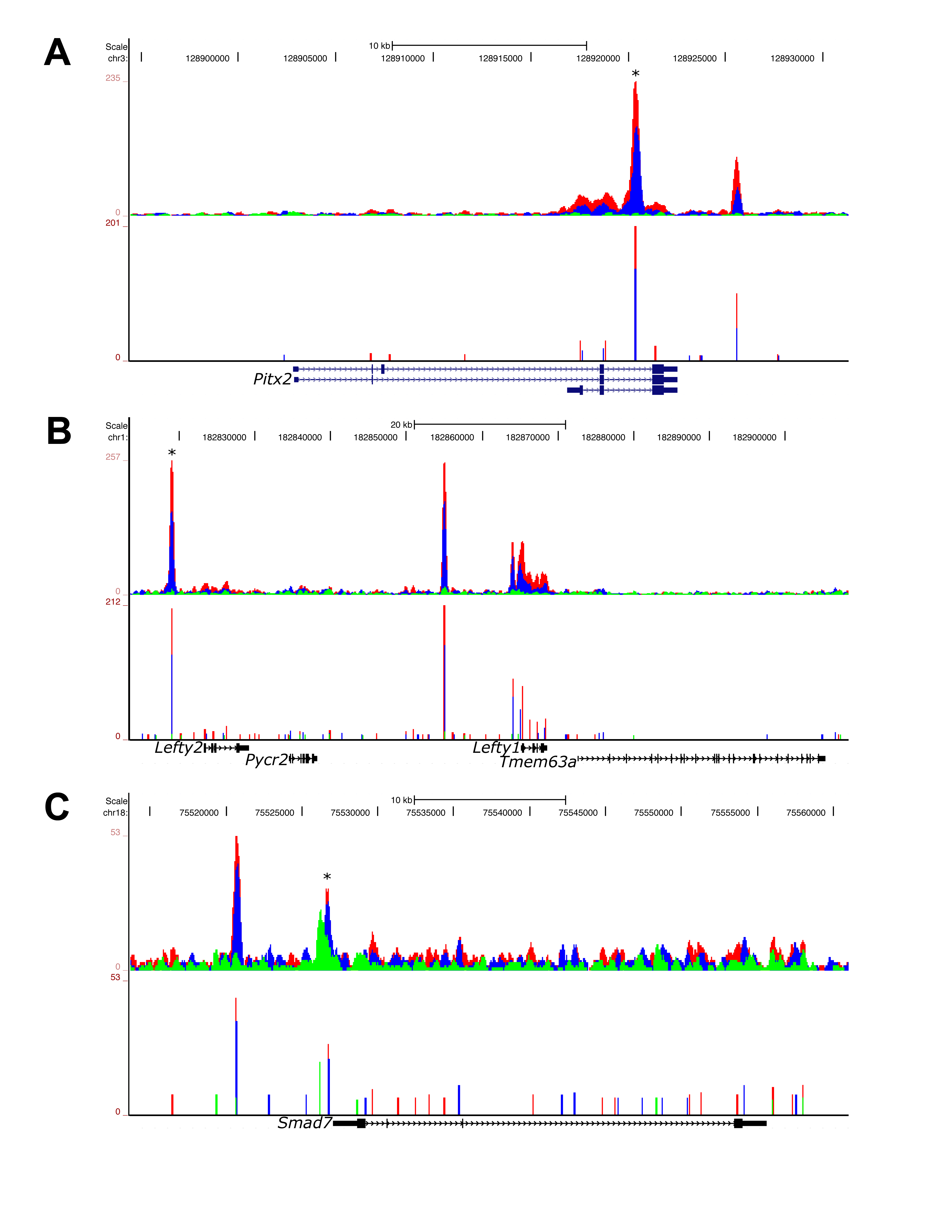

Supplement: Figure S3 — Pitx2, the Lefty1/2 Hotspot, and Smad7 Contain Multiple pSmad2 Regulatory Sites in Their Genetic Loci. UCSC Genome Browser representation of the genomic loci for Pitx2 (A) on chromosome 3:128,894,461–128,931,307, Lefty1/2, Pycr2 and Tmem63a (B) on chromosome 1:182,813,583–182,908,336 and Smad7 (C) on chromosome 18:75,513,703–75,561,010. Genomic coordinates are shown on the x-axis while raw pSmad2 ChIP-Seq tag counts are presented on the y-axis in the top panel followed by relative enrichments normalized to the respective input DNA controls of Activin (red), DMSO (blue) and SB (green) treated ES cells in the bottom panel. The RefSeq genetic structures of the indicated genes and their isoforms if any are indicated below the ChIP-Seq panels. Scale bar shows genomic distance in kilo base pairs (kb) while pSmad2 enrichment peaks corresponding to the real-time PCR results in Figure S1 are indicated with asterisks (*). (TIF) [file pgen.1002130.s003.tif]

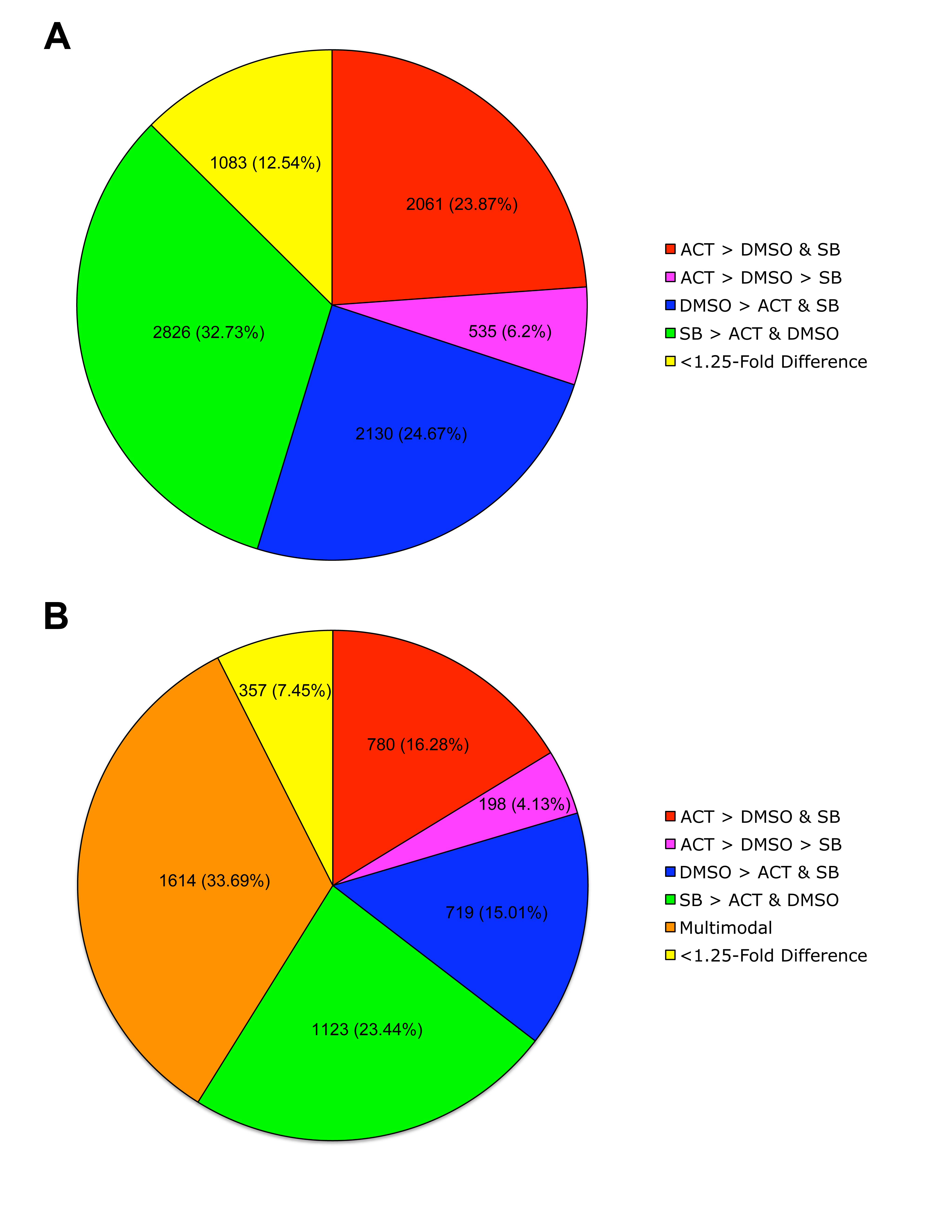

Supplement: Figure S4 — Phospho-Smad2 Binding to Majority of Sites and Target Genes Varies by >1.25-Fold during Graded Signaling. Pie charts showing patterns and statistics of pSmad2 binding to sites and target genes that vary by at least 1.25-fold during Activin, DMSO and SB treatments. Colored segments display the indicated models of pSmad2 binding in the 3 signaling conditions. Proportional segment sizes define the relative contribution of each type of binding behavior for ChIP-Seq peaks (A) and target genes within +/−50 kb of the peaks (B) out of the total for each. Values indicate the number of peaks (A) or genes (B) in each regulatory model with percentages of the total in parentheses. (TIF) [file pgen.1002130.s004.tif]

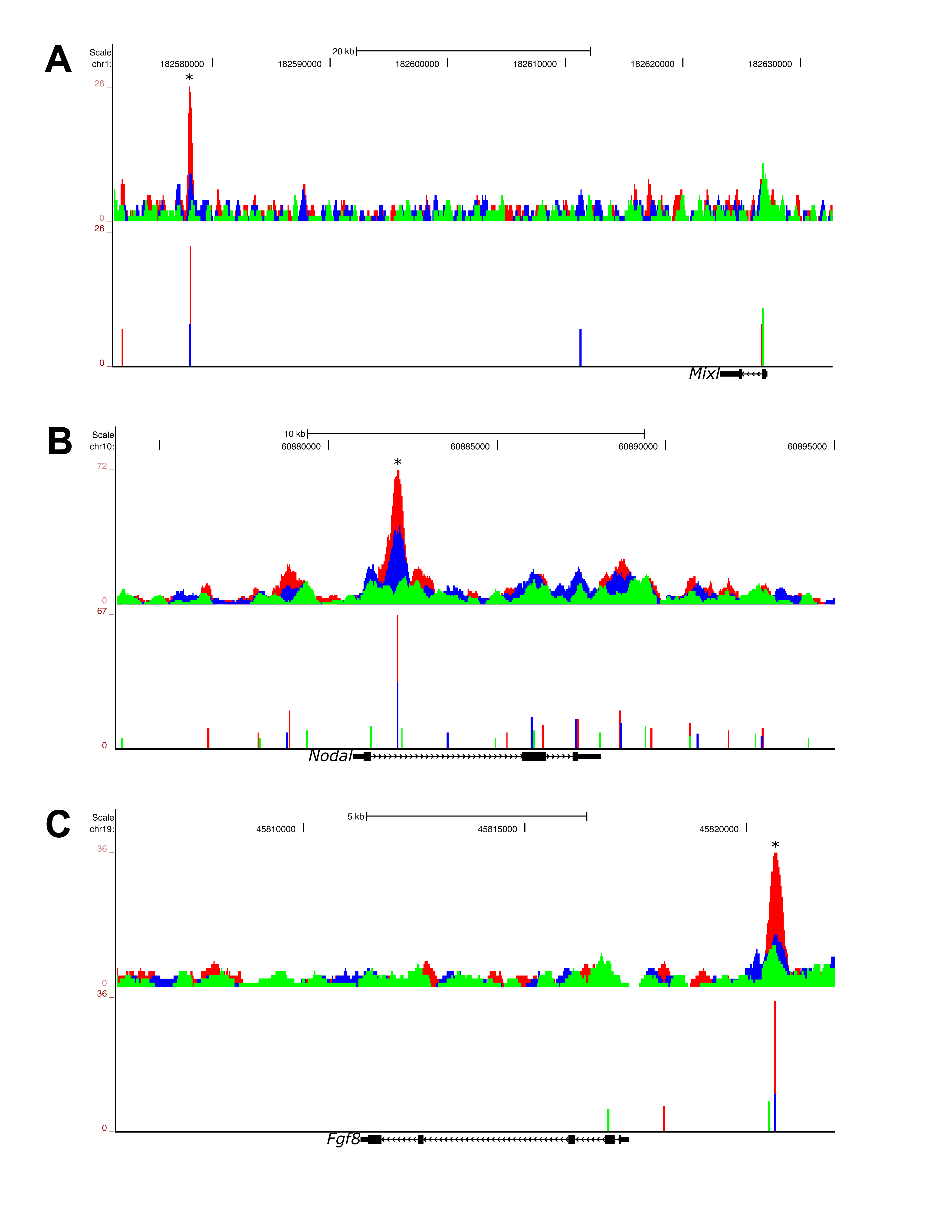

Supplement: Figure S5 — The Mesendodermal Genes Mixl, Nodal, and Fgf8 Are Targets of Differential pSmad2 Binding. Binding profile of pSmad2 ChIP-Seq enrichments on Mixl (A), Nodal (B) and Fgf8 (C) as visualized on the UCSC Genome Browser at the coordinates chr1:182,571,609–182,632,748, chr10:60,873,730–60,895,009 and chr19–45:805,804–45,822,014 respectively. Changes in the level of pSmad2 ChIP-Seq peak enrichments (raw peaks top panel, normalized peaks bottom panel) are indicated for Activin (red), DMSO (blue) and SB (green) treated ES cells on the y-axis. X-axis shows genomic location and genetic features of the indicated genes are below the Genome Browser panels. PSmad2 binding peaks showing significant differential binding with Nodal/Activin signaling are marked with asterisks (*). Scale bar shows genomic distance in kilo base pairs (kb). (TIF) [file pgen.1002130.s005.tif]

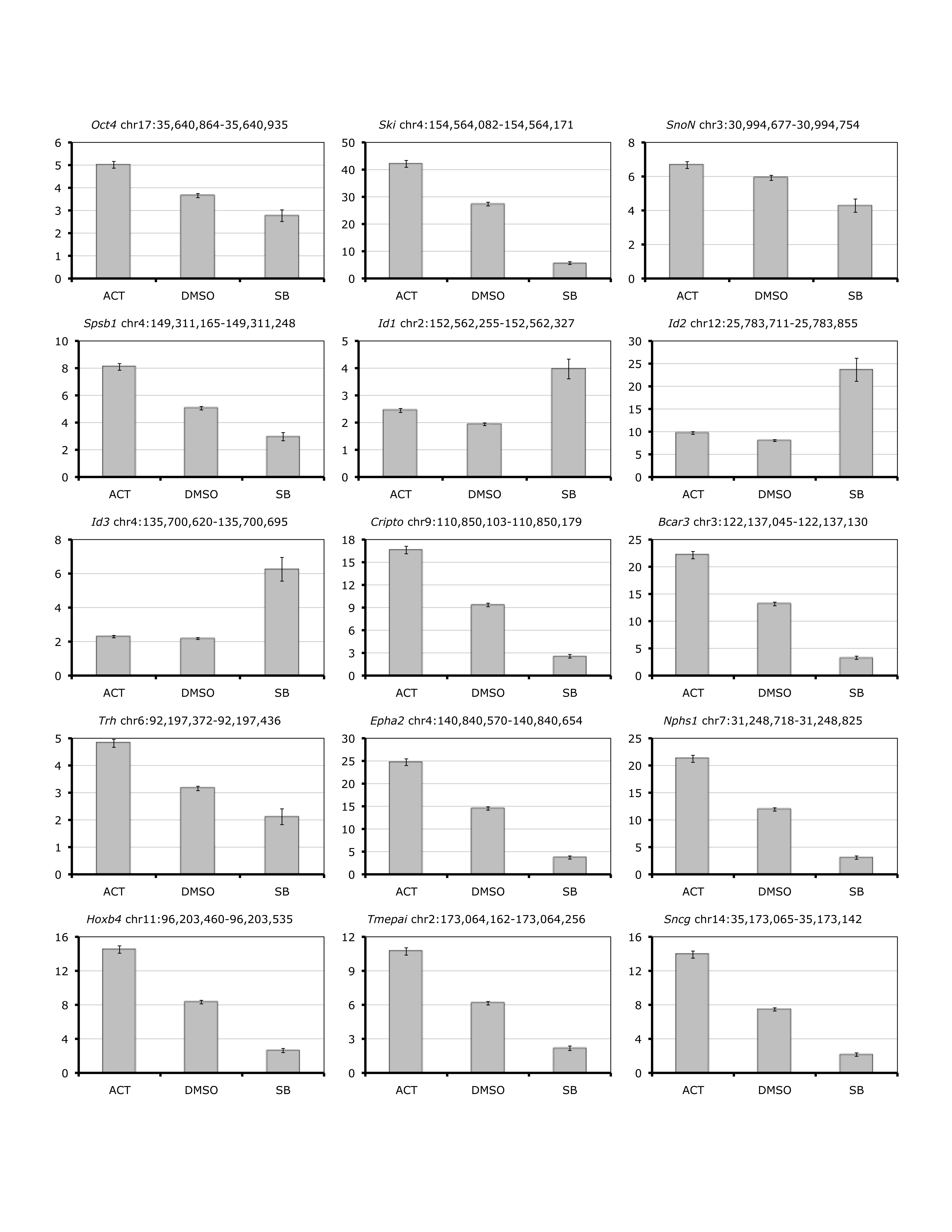

Supplement: Figure S6 — Validation of 15 Genes as Direct Targets of Nodal/Activin Signaling via Differential Phospho-Smad2 Binding. Real-time PCR validation of pSmad2 ChIP-Seq enrichment on original unamplified ChIP DNA from ES cells treated with Activin (ACT), DMSO and SB in KSR media for 18 hours. Indicated genes were selected based on transcriptional regulation under the same conditions in the microarray analysis of Figure 3A and real-time PCR gene expression detection in Figure 7D. Vertical scale shows fold-enrichment over the non-binding control region in the Pfkm locus on chromosome 15:97,934,552–97,934,626 after normalization to the input DNA controls in each treatment. Phospho-Smad2 enriched regions detected by the PCR primers are indicated in the genomic coordinates above each graph. Error bars show s.e.m. for n = 12 replicates. (TIF) [file pgen.1002130.s006.tif]
