# Supplementary material for: Graded Nodal/Activin Signaling Titrates Conversion of Quantitative Phospho-Smad2 Levels into Qualitative Embryonic Stem Cell Fate Decisions
Source: PLoS Genet. 2011 Jun 23;7(6):e1002130. doi: 10.1371/journal.pgen.1002130 (PMC3121749; doi:10.1371/journal.pgen.1002130)
Supplement: Table S1 — 64.2% of Microarray Targets Differentially Expressed by >1.5-fold are also Targets of Differential Phospho-Smad2 Binding. Table showing 104 out of the 162 microarray target genes from Figure 3A where expression is induced and/or repressed by >1.5-fold during stimulation or inhibition of Nodal/Activin signaling in ES cells. These targets also exhibit pSmad2 binding at +/−50 kb from the 5′/3′UTRs and within the genes themselves. Only ChIP-Seq peaks that satisfy a size cutoff of >12 sequencing tags in at least 1 condition are considered significantly above background sequencing levels and counted for each gene during Activin (ACT), DMSO vehicle control and SB inhibitor treatments. Gene IDs of the target genes, their chromosomal locations (Chr) and their orientation on the sense (+)/antisense (−) strands are as indicated. Gene names highlighted in yellow are for Activin/SB co-regulated targets, red for Activin specific targets and green for SB responsive genes only. The number of sequencing tags within the largest ChIP-Seq peaks for each gene and under each condition are as shown. For genes that contain multiple pSmad2 binding sites, the largest peaks counted may not occur at the same genomic location. Changes in the level of overall pSmad2 binding during Activin treatment compared to SB (ACT vs SB), Activin compared to DMSO control (ACT vs DMSO) and SB compared to DMSO (SB vs DMSO) are shown as (+) for increased binding and (–) for decreased binding by >1.5-fold. The overall level of pSmad2 binding for each gene is calculated as the sum of all peak heights (enrichment counts) proximal to the gene and under each condition. A value of 0 denotes no significant change in binding at a 1.5-fold cutoff and NA indicates no detectable peaks for statistical analysis. (DOC) [file pgen.1002130.s007.doc]

**Table S1**

| **Gene** | **Gene ID** | **Chr** | **Strand** | **No. Peaks ACT** | **No. Peaks DMSO** | **No. Peaks SB** | **Largest Peak ACT** | **Largest Peak DMSO** | **Largest Peak SB** | **ACT vs SB** | **ACT vs DMSO** | **SB vs DMSO** |
| --- | --- | --- | --- | --- | --- | --- | --- | --- | --- | --- | --- | --- |
| Lefty1 | NM_010094 | 1 | + | 6 | 7 | 2 | 824 | 313 | 33 | + | + | - |
| Lefty2 | NM_177099 | 1 | + | 7 | 7 | 2 | 824 | 313 | 33 | + | + | - |
| Pitx2 | NM_011098 | 3 | + | 4 | 5 | 0 | 745 | 287 | NA | + | + | - |
| Fgf15 | NM_008003 | 7 | + | 4 | 5 | 5 | 64 | 59 | 83 | - | 0 | 0 |
| Tmem63a | NM_144794 | 1 | + | 6 | 6 | 2 | 824 | 279 | 33 | + | + | - |
| Pycr2 | NM_133705 | 1 | + | 7 | 7 | 2 | 824 | 313 | 33 | + | + | - |
| Spsb1 | NM_029035 | 4 | - | 2 | 1 | 0 | 74 | 61 | NA | + | + | - |
| Chst15 | NM_029935 | 7 | - | 1 | 1 | 0 | 43 | 19 | NA | + | + | - |
| Radil | NM_178702 | 5 | - | 1 | 2 | 1 | 170 | 74 | 28 | + | + | - |
| Tubb6 | NM_026473 | 18 | + | 1 | 1 | 1 | 48 | 27 | 34 | 0 | + | 0 |
| Arhgap9 | NM_146011 | 10 | + | 2 | 5 | 4 | 65 | 32 | 81 | - | 0 | + |
| Zfp92 | NM_009566 | X | + | 0 | 0 | 1 | NA | NA | 18 | - | NA | + |
| Arhgef10l | NM_001112722 | 4 | - | 1 | 2 | 2 | 21 | 32 | 56 | - | - | + |
| Golga2 | NM_001080968 | 2 | + | 0 | 0 | 1 | NA | NA | 21 | - | NA | + |
| Zc3h3 | NM_172121 | 15 | - | 0 | 2 | 0 | NA | 14 | NA | NA | - | - |
| Gpbp1l1 | NM_029868 | 4 | + | 4 | 5 | 4 | 40 | 36 | 55 | 0 | 0 | 0 |
| Gdf15 | NM_011819 | 8 | - | 4 | 2 | 1 | 405 | 252 | 159 | + | + | - |
| Prf1 | NM_011073 | 10 | + | 0 | 0 | 1 | NA | NA | 19 | - | NA | + |
| Olfr1371 | NM_207253 | 11 | - | 0 | 1 | 1 | NA | 17 | 19 | - | - | 0 |
| Orai3 | NM_198424 | 7 | + | 2 | 4 | 2 | 69 | 53 | 103 | 0 | 0 | 0 |
| 2810474O19Rik | NM_026054 | 6 | + | 1 | 0 | 1 | 13 | NA | 15 | 0 | + | + |
| Utp15 | NM_178918 | 13 | - | 2 | 2 | 1 | 38 | 14 | 20 | + | + | 0 |
| Crk | NM_133656 | 11 | + | 6 | 4 | 2 | 29 | 45 | 44 | + | 0 | - |
| Tubd1 | NM_019756 | 11 | + | 1 | 0 | 0 | 13 | NA | NA | + | + | NA |
| Id1 | NM_010495 | 2 | + | 3 | 3 | 2 | 38 | 43 | 45 | 0 | 0 | 0 |
| Id2 | NM_010496 | 12 | - | 0 | 0 | 2 | NA | NA | 62 | - | NA | + |
| Id3 | NM_008321 | 4 | + | 0 | 1 | 2 | NA | 12 | 58 | - | - | + |
| Serping1 | NM_009776 | 2 | - | 2 | 6 | 1 | 411 | 183 | 486 | 0 | 0 | 0 |
| Slc30a2 | NM_001039677 | 4 | + | 1 | 1 | 0 | 33 | 9 | NA | + | + | - |
| Rbm20 | NM_001170847 | 19 | + | 0 | 1 | 1 | NA | 12 | 26 | - | - | + |
| Rgma | NM_177740 | 7 | + | 1 | 1 | 1 | 39 | 28 | 33 | 0 | 0 | 0 |
| Abi3 | NM_001163464 | 11 | - | 0 | 1 | 0 | NA | 20 | NA | NA | - | - |
| 1110021L09Rik | NM_183116 | 10 | + | 2 | 3 | 4 | 231 | 197 | 224 | 0 | 0 | 0 |
| Tcfap2c | NM_009335 | 2 | + | 0 | 0 | 1 | NA | NA | 19 | - | NA | + |
| Actr2 | NM_146243 | 11 | - | 2 | 1 | 1 | 24 | 12 | 21 | + | + | + |
| Fam178b | NM_001126046 | 1 | - | 0 | 1 | 4 | NA | 23 | 58 | - | - | + |
| Tsc1 | NM_022887 | 2 | + | 0 | 2 | 2 | NA | 13 | 43 | - | - | + |
| Dlgap3 | NM_198618 | 4 | + | 2 | 0 | 2 | 34 | NA | 70 | - | + | + |
| Elovl1 | NM_019422 | 4 | + | 2 | 1 | 2 | 31 | 34 | 44 | - | + | + |
| Limd1 | NM_013860 | 9 | + | 2 | 1 | 1 | 95 | 96 | 92 | 0 | 0 | 0 |
| Crabp2 | NM_007759 | 3 | + | 1 | 3 | 1 | 25 | 42 | 43 | - | - | - |
| Rnf216 | NM_080561 | 5 | - | 2 | 3 | 0 | 18 | 45 | NA | + | - | - |
| Tcfap2c | NM_009335 | 2 | + | 0 | 0 | 1 | NA | NA | 19 | - | NA | + |
| Itgal | NM_008400 | 7 | + | 3 | 2 | 2 | 49 | 41 | 73 | 0 | + | + |
| Hspb8 | NM_030704 | 5 | - | 1 | 1 | 0 | 19 | 17 | NA | + | 0 | - |
| Mt1 | NM_013602 | 8 | + | 1 | 0 | 0 | 38 | NA | NA | + | + | NA |
| Ston2 | NM_175367 | 12 | - | 0 | 4 | 2 | NA | 12 | 19 | - | - | 0 |
| Odf2 | NM_001113213 | 2 | + | 0 | 2 | 1 | NA | 35 | 15 | - | - | - |
| Tcfap2c | NM_009335 | 2 | + | 0 | 0 | 1 | NA | NA | 19 | - | NA | + |
| Parp6 | NM_029922 | 9 | + | 7 | 13 | 4 | 561 | 252 | 542 | 0 | 0 | 0 |
| Rusc1 | NM_028188 | 3 | - | 1 | 2 | 2 | 30 | 56 | 62 | - | - | 0 |
| Nme4 | NM_019731 | 17 | - | 0 | 4 | 1 | NA | 55 | 42 | - | - | - |
| Chn2 | NM_001163640 | 6 | + | 1 | 0 | 0 | 15 | NA | NA | + | + | NA |
| Zscan5b | NM_133204 | 7 | + | 0 | 0 | 2 | NA | NA | 72 | - | NA | + |
| Fn1 | NM_010233 | 1 | - | 1 | 4 | 1 | 17 | 20 | 29 | - | - | - |
| Cyp7b1 | NM_007825 | 3 | - | 1 | 0 | 0 | 19 | NA | NA | + | + | NA |
| Stmn3 | NM_009133 | 2 | - | 2 | 1 | 1 | 31 | 17 | 14 | + | + | 0 |
| Vps25 | NM_026776 | 11 | + | 1 | 1 | 1 | 38 | 33 | 40 | 0 | 0 | 0 |
| Ecm1 | NM_007899 | 3 | - | 1 | 1 | 0 | 41 | 12 | NA | + | + | - |
| Tacc2 | NM_206856 | 7 | + | 1 | 0 | 1 | 22 | NA | 42 | - | + | + |
| Copz2 | NM_019877 | 11 | + | 5 | 8 | 4 | 44 | 47 | 136 | - | - | 0 |
| Cripto | NM_011562 | 9 | - | 5 | 7 | 5 | 161 | 126 | 53 | + | + | - |
| Bcar3 | NM_013867 | 3 | + | 2 | 1 | 0 | 89 | 58 | NA | + | + | - |
| Ceacam18 | NM_028236 | 7 | + | 1 | 2 | 1 | 36 | 14 | 19 | + | 0 | 0 |
| Fam19a4 | NM_177233 | 6 | - | 1 | 1 | 0 | 34 | 20 | NA | + | + | - |
| Rprm | NM_023396 | 2 | - | 1 | 0 | 0 | 21 | NA | NA | + | + | NA |
| Trh | NM_009426 | 6 | - | 2 | 1 | 0 | 26 | 23 | NA | + | + | - |
| Mpp7 | NM_001081287 | 18 | - | 1 | 0 | 0 | 18 | NA | NA | + | + | NA |
| Il17rd | NM_134437 | 14 | + | 1 | 1 | 1 | 26 | 14 | 51 | - | + | + |
| Tnfrsf12a | NM_001161746 | 17 | - | 1 | 0 | 2 | 23 | NA | 38 | - | + | + |
| Epha2 | NM_010139 | 4 | + | 15 | 16 | 4 | 195 | 153 | 60 | + | + | - |
| Gm1631 | NM_201366 | 2 | + | 1 | 0 | 0 | 47 | NA | NA | + | + | NA |
| Nphs1 | NM_019459 | 7 | + | 4 | 3 | 0 | 158 | 81 | NA | + | + | - |
| 2210011C24Rik | AK008705 | 8 | - | 3 | 4 | 2 | 74 | 50 | 83 | 0 | 0 | 0 |
| Ubr7 | NM_025666 | 12 | + | 2 | 4 | 0 | 126 | 72 | NA | + | + | - |
| Hoxb4 | NM_010459 | 11 | + | 1 | 2 | 2 | 83 | 71 | 49 | 0 | 0 | 0 |
| Bhlha15 | NM_010800 | 5 | + | 0 | 0 | 1 | NA | NA | 14 | - | NA | + |
| Ccdc137 | NM_152807 | 11 | + | 0 | 2 | 2 | NA | 41 | 99 | - | - | + |
| Bcl11b | NM_021399 | 12 | - | 0 | 0 | 1 | NA | NA | 29 | - | NA | + |
| Lrrfip2 | NM_027742 | 9 | + | 2 | 2 | 0 | 20 | 9 | NA | + | + | - |
| Thnsl2 | NM_178413 | 6 | - | 0 | 1 | 0 | NA | 16 | NA | NA | - | - |
| Plekha2 | NM_031257 | 8 | - | 2 | 1 | 0 | 89 | 53 | NA | + | + | - |
| Tmepai | NM_022995 | 2 | - | 2 | 2 | 2 | 116 | 59 | 28 | + | 0 | - |
| Fhod3 | NM_175276 | 18 | + | 1 | 1 | 1 | 37 | 41 | 21 | + | 0 | - |
| Slc7a7 | NM_011405 | 14 | - | 3 | 6 | 0 | 72 | 29 | NA | + | 0 | - |
| Cdh3 | NM_001037809 | 8 | + | 0 | 1 | 0 | NA | 58 | NA | NA | - | - |
| Nop16 | NM_178605 | 13 | - | 1 | 0 | 1 | 16 | NA | 32 | - | + | + |
| Raet1a | NM_009016 | 10 | + | 1 | 0 | 0 | 35 | NA | NA | + | + | NA |
| Pex11c | NM_026951 | 8 | - | 3 | 4 | 5 | 109 | 71 | 83 | 0 | 0 | 0 |
| Ccnd2 | NM_009829 | 6 | - | 0 | 1 | 0 | NA | 18 | NA | NA | - | - |
| Tnfsf13 | NM_023517 | 11 | - | 5 | 4 | 2 | 272 | 356 | 308 | 0 | 0 | 0 |
| Ensa | NM_019561 | 3 | + | 11 | 11 | 5 | 435 | 314 | 417 | + | 0 | 0 |
| Slc4a5 | NM_001166067 | 6 | + | 0 | 1 | 0 | NA | 18 | NA | NA | - | - |
| Sncg | NM_011430 | 14 | - | 4 | 2 | 3 | 86 | 65 | 45 | + | + | 0 |
| Lef1 | NM_010703 | 3 | + | 1 | 0 | 0 | 17 | NA | NA | + | + | NA |
| Notch4 | NM_010929 | 17 | + | 1 | 2 | 1 | 23 | 35 | 25 | 0 | - | - |
| Egln3 | NM_028133 | 12 | - | 3 | 1 | 2 | 105 | 52 | 25 | + | + | 0 |
| Wiz | NM_011717 | 17 | - | 1 | 2 | 1 | 11 | 58 | 71 | - | - | 0 |
| Klhl8 | NM_178741 | 5 | - | 2 | 1 | 0 | 25 | 16 | NA | + | + | - |
| 7420416P09Rik | NM_001168589 | 12 | + | 1 | 0 | 0 | 58 | NA | NA | + | + | NA |
| Timm50 | NM_025616 | 7 | - | 1 | 2 | 4 | 61 | 88 | 77 | - | - | 0 |
| Fhod1 | NM_177699 | 8 | - | 1 | 2 | 1 | 93 | 49 | 45 | + | 0 | - |
| Gstcd | NM_026231 | 3 | - | 0 | 0 | 1 | NA | NA | 29 | - | NA | + |
| Tmem220 | NM_177392 | 11 | + | 1 | 0 | 0 | 13 | NA | NA | + | + | NA |
