# Supplementary material for: Graded Nodal/Activin Signaling Titrates Conversion of Quantitative Phospho-Smad2 Levels into Qualitative Embryonic Stem Cell Fate Decisions
Source: PLoS Genet. 2011 Jun 23;7(6):e1002130. doi: 10.1371/journal.pgen.1002130 (PMC3121749; doi:10.1371/journal.pgen.1002130)
Supplement: Table S2 — There Are Dynamic Changes in Phospho-Smad2 Binding Motifs During Graded Nodal/Activin Signaling. MotifEnrich program identification of co-motifs enriched within ≤5000 bp of pSmad2 binding peaks. Co-motifs are ranked based on frequency of occurrence from highest to lowest. Matching of co-motifs with associated transcription factors was performed using the TRANSFAC PWM database with p-value thresholds of 1e-05. Transcription factor names in black are present across all 3 signaling conditions with Activin, DMSO and SB treatments. Activin enriched transcription partners binding on the co-motifs in proximity to pSmad2 sites are highlighted in red, DMSO enriched factors in blue and SB in green. The intervals of significant enrichment for each co-motif are indicated in base pairs (bp). (DOC) [file pgen.1002130.s008.doc]

**Table S2**

| **Rank**  **(Activin)** | **Family** | **TRANSFAC PWM** | **Interval**  **(bp)** | **Rank**  **(DMSO)** | **Family** | **TRANSFAC PWM** | **Interval**  **(bp)** | **Rank**  **(SB)** | **Family** | **TRANSFAC PWM** | **Interval**  **(bp)** |
| --- | --- | --- | --- | --- | --- | --- | --- | --- | --- | --- | --- |
| 1 | **E2F** | V_E2F1_Q3_01 | 998 | 1 | **ETS** | V_ELK1_02 | 743 | 1 | **NRF** | V_NRF1_Q6 | 1000 |
| 2 | **SP1** | V_GC_01 | 998 | 2 | **HIC1** | V_HIC1_02 | 964 | 2 | **AP2** | V_AP2_Q3 | 990 |
| 3 | **AP2** | V_AP2_Q6_01 | 896 | 3 | **AP2** | V_AP2_Q3 | 999 | 3 | **E2F** | V_E2F1_Q3_01 | 1000 |
| 4 | **HIC1** | V_HIC1_03 | 1000 | 4 | **E2F** | V_E2F1_Q3_01 | 999 | 4 | **HIC1** | V_HIC1_02 | 999 |
| 5 | **ETS** | V_ELK1_02 | 920 | 5 | **DEAF1** | V_DEAF1_01 | 978 | 5 | **EGR** | V_EGR3_01 | 997 |
| 6 | **WHN** | V_WHN_B | 932 | 6 | **NRF** | V_NRF1_Q6 | 949 | 6 | **MTF1** | V_MTF1_Q4 | 990 |
| 7 | **DEAF1** | V_DEAF1_01 | 932 | 7 | **AHR** | V_AHRARNT_02 | 987 | 7 | **DEAF1** | V_DEAF1_01 | 1000 |
| 8 | **ZF5** | V_ZF5_B | 1000 | 8 | **EGR** | V_EGR3_01 | 988 | 8 | **AHR** | V_AHRARNT_02 | 949 |
| 9 | **NRF** | V_NRF1_Q6 | 970 | 9 | **MINI** | V_MINI20_B | 993 | 9 | **SP1** | V_GC_01 | 959 |
| 10 | **MINI** | V_MINI20_B | 998 | 10 | **SP1** | V_SP1_Q6_01 | 1000 | 10 | **PAX** | V_PAX4_01 | 995 |
| 11 | **PAX** | V_PAX5_01 | 783 | 11 | **EBOX** | V_E47_01 | 748 | 11 | **ETS** | V_GABP_B | 979 |
| 12 | **EBOX** | V_HIF1_Q5 | 998 | 12 | **WHN** | V_WHN_B | 737 | 12 | **ZF5** | V_ZF5_B | 999 |
| 13 | **EGR** | V_EGR3_01 | 999 | 13 | **ZF5** | V_ZF5_B | 752 | 13 | **EBOX** | V_MYCMAX_B | 998 |
| 14 | **OCT** | V_OCT4_02 | 149 | 14 | **PAX** | V_PAX5_01 | 991 | 14 | **WHN** | V_WHN_B | 988 |
| 15 | **NFKB** | V_NFKB_Q6_01 | 276 | 15 | **CREB** | V_CREB_02 | 940 | 15 | **R** | V_R_01 | 854 |
| 16 | **AHR** | V_AHRARNT_02 | 966 | 16 | **R** | V_R_01 | 883 | 16 | **CREB** | V_ATF_01 | 996 |
| 17 | **MOVO** | V_MOVOB_01 | 996 | 17 | **MTF1** | V_MTF1_Q4 | 963 | 17 | **MINI** | V_MINI20_B | 958 |
| 18 | **STAT** | V_STAT3_02 | 157 | 18 | **SP3** | V_SP3_Q3 | 997 | 18 | **NFKB** | V_NFKAPPAB50_01 | 999 |
| 19 | **R** | V_R_01 | 985 | 19 | **CAAT** | V_YY1_Q6_02 | 390 | 19 | **MOVO** | V_MOVOB_01 | 999 |
| 20 | **MTF1** | V_MTF1_Q4 | 995 | 20 | **HES** | V_HES1_Q2 | 409 | 20 | **HES** | V_HES1_Q2 | 995 |
| 21 | **ROAZ** | V_ROAZ_01 | 182 | 21 | **ZNF219** | V_ZNF219_01 | 60 | 21 | **HEN** | V_HEN1_02 | 954 |
| 22 | **CREB** | V_ATF_01 | 366 | 22 | **GGG** | V_CHCH_01 | 946 | 22 | **GGG** | V_CHCH_01 | 994 |
| 23 | **ERE** | V_SF1_Q6 | 117 | 23 | **HEN** | V_HEN1_01 | 328 | 23 | **LRF** | V_LRF_Q2 | 984 |
| 24 | **GGG** | V_CHCH_01 | 943 | 24 | **E2** | V_E2_01 | 576 | 24 | **CAAT** | V_CAAT_C | 295 |
| 25 | **SMAD** | V_SMAD4_Q6 | 280 | 25 | **MOVO** | V_MOVOB_01 | 944 | 25 | **IK** | V_IK1_01 | 897 |
| 26 | **KAISO** | V_KAISO_01 | 182 | 26 | **NFKB** | V_NFKAPPAB50_01 | 986 | 26 | **PLZF** | V_PLZF_02 | 71 |
| 27 | **HEN** | V_HEN1_02 | 640 | 27 | **AP4** | V_AP4_Q6_01 | 826 |  |  |  |  |
| 28 | **LMAF** | V_LMAF_Q2 | 271 | 28 | **CP2** | V_CP2_02 | 877 |  |  |  |  |
| 29 | **E2** | V_E2_Q6 | 601 |  |  |  |  |  |  |  |  |
| 30 | **CACCC** | V_CACBINDINGPROTEIN_Q6 | 637 |  |  |  |  |  |  |  |  |
| 31 | **CAAT** | V_NFY_01 | 875 |  |  |  |  |  |  |  |  |
| 32 | **P53** | V_P53_01 | 143 |  |  |  |  |  |  |  |  |
| 33 | **AP4** | V_AP4_01 | 157 |  |  |  |  |  |  |  |  |
| 34 | **IPF** | V_IPF1_Q4_01 | 502 |  |  |  |  |  |  |  |  |
| 35 | **VMYB** | V_VMYB_02 | 915 |  |  |  |  |  |  |  |  |
| 36 | **SOX** | V_SOX9_B1 | 65 |  |  |  |  |  |  |  |  |
| 37 | **RFX** | V_RFX_Q6 | 172 |  |  |  |  |  |  |  |  |
| 38 | **SZF11** | V_SZF11_01 | 118 |  |  |  |  |  |  |  |  |
| 39 | **STAF** | V_STAF_02 | 558 |  |  |  |  |  |  |  |  |
| 40 | **ZID** | V_ZID_01 | 82 |  |  |  |  |  |  |  |  |
| 41 | **CP2** | V_CP2_02 | 137 |  |  |  |  |  |  |  |  |
| 42 | **GLI** | V_ZIC3_01 | 115 |  |  |  |  |  |  |  |  |
