# Supplementary material for: Graded Nodal/Activin Signaling Titrates Conversion of Quantitative Phospho-Smad2 Levels into Qualitative Embryonic Stem Cell Fate Decisions
Source: PLoS Genet. 2011 Jun 23;7(6):e1002130. doi: 10.1371/journal.pgen.1002130 (PMC3121749; doi:10.1371/journal.pgen.1002130)
Supplement: Table S3 — Primers Used for Quantification of ChIP DNA and Marker/Gene Expression Levels by Real-Time PCR. List of forward/reverse primer pairs used in SYBR Green real-time PCR reactions for the quantification of ChIP-DNA (ChIP-qPCR) and Marker/Gene expression. For tiling ChIP-qPCR analysis of pSmad2 binding on Pitx2, Lefty2 and Smad7, primers for each gene are numbered sequentially according to the 5′–3′ order of their respective amplified regions shown in Figure S1. (DOC) [file pgen.1002130.s009.doc]

Table S3

| **ChIP-qPCR Primers** | **Forward** | **Reverse** |
| --- | --- | --- |
| Bcar3 | CCCATCTGTGGAGGAGTGACTT | CACTCTCAGTCTCCTTTGTTATTCAAGA |
| Cripto | CCTGTAGGGATCAGAAAGACCAGAT | AAAGGTGTCGCCCTCCTAATC |
| Epha2 | GAGGGAATTTGTCCCCTGATG | CCCATTGTTGTCTGGGCTTT |
| Hoxb4 | GAGGCTCTCCGAATCCACAA | AGACGTGATAGGAAAGAATGGAGACT |
| Id1 | ACGACATGAACGGCTGCTACT | TTGCTCACTTTGCGGTTCTG |
| Id2 | CCCGGAGCAGACTCTCTGG | TTGCAGGCATTGATCAGCTG |
| Id3 | TCCGCCTGTGGTCCTTTG | CGGTCTCTACCTCCAGCTGATAA |
| Lefty2 1 | AGAAAACCCAAAACAGATCTCAGG | ACTACCTCTGACGCAAAGAAAGAAA |
| Lefty2 2 | GCGTCAGAGGTAGTCAGGAAGC | AGCAGTCAGTCCAGTGAGACAAAT |
| Lefty2 3 | CTGGACCCTGTCTCTAGTGAAATCT | GCCTACTGTTCTGTCACTCTCTACCA |
| Lefty2 4 | CCAACCCTGAAGCACGGA | CCCTGTTCGCTCCATCCTC |
| Lefty2 5 | GAACATTCGCAGCATAGAAGCTC | TCTCCCCCAAGGGCTTCTAA |
| Lefty2 6 | CTTTCTAGGACCGGAGGTGCT | AGCTAGGCCTCTCCCTCATATTG |
| Lefty2 7 | TCACAAAAGTAGCCCCACCCT | GCCCAAAGAAGGAAGCAGATG |
| Lefty2 8 | CCAATCACTGTATTTCCCCAACA | AAACGCAGACAGAAAAACAGAGG |
| Lefty2 9 | GCTGCTGATGGATGGCATCT | CACACGGGACTGATACACAGACA |
| Lefty2 10 | TCCGAGACTGTTTTCTCTGTACCA | AACGCCCCATCATCATCG |
| Lefty2 11 | ATCACCTATGCACGCCCCTAG | CTGGGAGTCGCTTTAGGGACT |
| Lefty2 12 | TGGCTCAGCGGTTATGATCA | CATCGGATCCTATTACAGATGGTTG |
| Lefty2 13 | AAAGCAGAGGACGGATTCAGAA | CTGGGTAGAAGAGGAACTGCATAGT |
| Lefty2 14 | TCCTAGGCTTAGCTATTCTATTGCTGA | TCAGTAAGACCTTCTGTGCCAGAG |
| Lefty2 15 | GCAGGGCCTTGGGTGTTAC | CCTTGCAATGGGCGAAAG |
| Lefty2 16 | GGTGAACTCTTGCATATTTAAGGCA | AACGGCACAGAGGTGTCACA |
| Nphs1 | GAGCTCACCCTGAGGATCCA | TCCCTCAATCCACAGCTTCTG |
| Oct4 | CATACTTGAACTGTGGTGGAGAGTG | TCCCCACACCCAGTTCCTC |
| Pfkm | GCCCAGCAAACACGGAAA | GCCAGCTTGCCTTAGGTATCC |
| Pitx2 1 | CTCCAGAAACACGCAAGGCT | GGTAAAGACCAGCGAGCAACTC |
| Pitx2 2 | CAGCCATTCAGATTTCTCTCACA | CACTGAGCTGAACTTTCTTGAGAAGA |
| Pitx2 3 | TGAGCTTAAACACATTACCCAGTAGC | AGTCTGCTGAGTCCCTCTTTCAGA |
| Pitx2 4 | AAGACGCAGTCAGTGTAGCTCTGA | GGCTCCGGGCAGTTAAACTAA |
| Pitx2 5 | GGCCTGGAGTAGAAATGACAAGTAA | CGTTTGGAAGTAGGCCTTTGG |
| Pitx2 6 | GGGCGGAATAGGAAATCAAGA | GGGCTGACAACGGTGATGTT |
| Pitx2 7 | GGAAAAAAGCGGATTAGATGGC | AGCCAGGCCTCTCTCCAATAC |
| Pitx2 8 | AGCCAGGCCTCTCTCCAATAC | GAGTGTGGATTGGAAGAGGGG |
| Pitx2 9 | CTCCAATACACAAAAGCTGCTCTC | AGTGTGGATTGGAAGAGGGGT |
| Pitx2 10 | GCCGAGTGTGGATTGGAAGA | CCTTCGTTTTGATCTGTATCACCTT |
| Pitx2 11 | CCGCAAATCCACAAGACAGA | AGCTGGCTAGGTTTGGGTAAGA |
| Pitx2 12 | TGGTTCAGAAATCCCAGTTTACC | CCCGGATGCCCTAGAAAAAT |
| Pitx2 13 | GACGAGGTGGTCTTTTGCTTTT | TACCGACTCTCCTCCCACACA |
| Pitx2 14 | AAGAGGGCCTAGAGGATCAAGG | CAATAGCTAAAAGGCCCTGGTG |
| Pitx2 15 | GCAAGAGTCCATGTGCATGC | GGGCACAGGGACAATGAGTT |
| Pitx2 16 | AGTTCAACGGGCTCATGCAG | AAGCTCTTGGTGGACAGAGACG |
| Pitx2 17 | TGAGTATGTCGTCCAGCATGG | CATGTGTCCCTATAAACGTACGGA |
| Pitx2 18 | TGAGTGCTTGCCAGTATGCAG | CTTCTAGCACAATTCTCAGTCTTTCTG |
| Ski | GGATGGTAGGGAACAAATTGCA | CACTGGAAGAAAGGCCACAGA |
| Smad7 1 | CTAAATATGTCAGGTTGGATCACCA | GTCAAGCAAAAGTCCGTGGAG |
| Smad7 2 | CCACGGACTTTTGCTTGACA | CAGAGAGTTTATTCCTTCACCATAGGT |
| Smad7 3 | AGCTGTGCACAAGACACACTTCTC | ACATTTACACCGGCCAGGAC |
| Smad7 4 | CGATCTCCTCCCCTTTCCAA | TTAGTGGCCCGATTTAGACCA |
| Smad7 5 | GGTCTAAATCGGGCCACTAACC | TCCCAGTTACTGCCACCCTCT |
| Smad7 6 | GGAGGGGGGTTGAAATAGCT | TCACGTGGCCGTCTAGACAC |
| Smad7 7 | GTGTCTAGACGGCCACGTGA | TCCCTCTGCTCGGCTGG |
| Smad7 8 | CGAGCCCTGGGCACATT | GTCTCAGGCAGCTCTCTCCG |
| Smad7 9 | GGCGCGACGAAGAGAGTCT | CTCTCATGAGCTGCTGGCC |
| Smad7 10 | CAGCATCTTCTGTCCCTGCTTC | TCTTGCGTATCTGGGTGTTTTAGA |
| Sncg | GGTGGCCCACACCTCTAATCT | GGAGCTTGTGTCTTTGAATGTCTAAT |
| SnoN | ACTTCCTCTGCCCCCTCTCA | CCCACTCATCATCAACCCAAA |
| Sox2 | CGGAATGGTTGGCGAGTGGTTAAACAGAGC | CGGAATGGTTGGCGAGTGGTTAAACAGAGC |
| Spsb1 | CCACTGCTAGCATCAAAGAGTGA | CCCGTGTAGCCAGCACTGA |
| Tmepai | CTGCACGAGGCCCTGTCT | CAGGCCTAACGAGATTAGATTTTCA |
| Trh | CCACTGCAGGTGTGCAAAGT | CGCCAGGCTTGTTGATTGT |
| **Marker/Gene Expression Primers** | **Forward** | **Reverse** |
| Actin | ACCAACTGGGACGACATGGAG | TACGACCAGAGGCATACAGGG |
| Bcar3 | GCAGTTGAAGCCCTTTAGCAA | GGCATCAGCAGAGGGACTGA |
| Cad11 | CGGCAAAGATTTCAGTAGAAGATGCCG | GCATCTGGGTCTTTGGCATGTA |
| Cdh3 | GCCAGGACTCTGAAGTTTGC | CAAGTTCAAGCCCTGAGAGG |
| Cdh3 | CATCCCCACCCCCATGTAC | AGTGTTGGCAGCCTTCAGGTT |
| Cdx2 | AGGCTGAGCCATGAGGAGTA | CGAGGTCCATAATTCCACTCA |
| Cer1 | CGAAGAGGTCTCCCAGTGTACTTCG | CCGTGACTCAGCCAGCAGAT |
| Claudin6 | CGAGACAAGATAGGAACTCCAAGTCTCG | ATAGAGTGGGCAGTCCAGCAGA |
| Dlx3 | GCCTTAGGGGTAAGGCTGTC | GACCTGCTTCTCTTGGTTGC |
| Ecad | CGGATGGTCTTTGTTCTGGTTATCCG | TGACGCAGCTCAAGAATCTCTCA |
| Eomes | CCTGGTGGTGTTTTGTTGTG | TTTAATAGCACCGGGCACTC |
| Errb | TTTCTGGAACCCATGGAGAG | AGCCAGCACCTCCTTCTACA |
| Esx | CTAACCCCAACCCCAACC | TTCTTGACAGGTAATGCGTGA |
| Fgf15 | CAGCAGCGTGCGGTACCT | AACAGTCCATTTCCTCCCTGAA |
| Fgf5 | ACTCCATGCAAGTGCCAAAT | CACTCTCGGCCTGTCTTTTC |
| Fgf8 | CGCTTTAGTTGAGGAACTCGAAGCG | ACATGGCCTTTACCCGCAAG |
| Flk1 | AAGTGACTTGCCCAGCATCT | CCGGTTCCCATCTCTCAGTA |
| Foxa2 | CGGGGTATGTCTTGGGGTCCCG | GGGCATGGGACCTCACCT |
| Gata3 | GGTTGTAGGCAAATCATTTG | CAACCAAAAATCCAGAGAGA |
| Gata4 | CTGGAAGACACCCCAATCTC | GTAGTGTCCCGTCCCATCTC |
| Gata6 | CTACACAAGCGACCACCTCA | TGTAGAGGCCGTCTTGACCT |
| Gfap | AAGCCAAGCACGAAGCTAAC | GAGCAAGTGCCTCCTGGTAA |
| Gsc | CGATTCTGTCCGAGTCCAAATCG | GGAGACGACAGAAGCGATCCTC |
| Hand1 | CCCCTCTTCCGTCCTCTTAC | CTGCGAGTGGTCACACTGAT |
| Hhex | CGAGACGGAGAGGTATTTCTGAGTCTCG | ACGACTACACGCACGCCCTAC |
| Id1 | ACGAGCAGCAGGTGAACGT | CCAGCTGCAGGTCCCTGAT |
| Id2 | CGCTGACCACCCTGAACAC | TCGACATAAGCTCAGAAGGGAAT |
| Id3 | ATCTCCAAGGACAAGAGGAGCTT | TGAAGAGGGCTGGGTTAAGATC |
| Itga7 | TTCCCCATACAATGCTGTGA | TAAAGGGCCCTGAGACAGTG |
| Lefty1 | TCGATCAACCGCCAGTCCTG | TGGGGACAGCCTCTTTTGCC |
| Lefty2 | GAACAGGTCCTGAGCAGTCTACTG | CCTCTCGAAAATTCTGGCTGAA |
| Mixl1 | CGTCTATGGTCTGTCGGAAGACG | CCACGCAGTGCTTTCCAAAC |
| Msi1 | AGGCTCTCACCCCTGGAC | CTGCCCCGTAGAGCTCAG |
| Nestin | GATCGCTCAGATCCTGGAAG | AGGTGTCTGCAAGCGAGAGT |
| Neurod1 | GCCTTTACCATGCACTACCC | TGTTGTCTATGGGGATCTCG |
| Ngn1 | GGAGTCGTCGCGTCAAAG | CAGGGCCCAGATGTAGTTGT |
| Nkx2.2 | TCTACGACAGCAGCGACAAC | TTGTCATTGTCCGGTGACTC |
| Nodal | CGGTTCTCATGCTCTACTCCAACCG | GGCTTCTGTCTGGCAAATGATG |
| Nphs1 | GGACCCCTCTATGATGAAGTACAAA | GATTCCCCTTGGGTCCTCATAT |
| Oct4 | ACCTTCAGGAGATATGCAAATCG | TTCTCAATGCTAGTTCGCTTTCTCT |
| Otx2 | CGCCTCCAAACAACCTTAGC | GGTGGCTGCAGGACAAGAA |
| Pax6 | AGTGAATGGGCGGAGTTATG | ACTTGGACGGGAACTGACAC |
| Pdgfra | CGCTCCTTCTACCACCTCAGCG | GCCGGATGGTCACTCTTTAGGA |
| Pdgfrb | CGGCCTCTGTTCTCTACACTGCCG | CCCTCTGGGAGACCTTCATCAG |
| Pitx2 | ACCCCGGCTATTCGTACAAC | GAGGACAGGGGATTGACGTTC |
| Serping1 | CTGGACAGCCTGCCTTCTG | TGGTTCAAATGTTATCTTCCACTTG |
| Smad7 | GGCCGGATCTCAGGCATTC | TTGGGTATCTGGAGTAAGGAGG |
| Sox1 | CTACGGCGGCATCCCTTAC | GGCTCCGACTTGACCAGAGA |
| Sox17 | CTGCACAACGCAGAGCTAAG | TTGTAGTTGGGGTGGTCCTG |
| Sox7 | CGGTACGATTACCCCAACTACAAGTACCG | GTCACGAGAGAGGGAGCTGAGG |
| Spsb1 | ATCCGCATGCGCTACTTGA | ATTTGGATCACTGGTAGAGGAGGTA |
| T | CGCTTGTTAGTTAGCTCCTTGAAGCG | CACAGAGAGCGCAGGGAAGAG |
| Tbx6 | CGGCCTACCTTCTACACCCTGCCG | CAGGCTGTAGGTCCAGAAATGC |
| Tdgf1 | CAGCTCCACTGTCTTCCTCAGA | GGAGTCCCGGATGCTTTGA |
| Ywhaz | CGTTGTAGGAGCCCGTAGGTCAT | TCTGGTTGCGAAGCATTGGG |
