# Supplementary material for: Graded Nodal/Activin Signaling Titrates Conversion of Quantitative Phospho-Smad2 Levels into Qualitative Embryonic Stem Cell Fate Decisions
Source: PLoS Genet. 2011 Jun 23;7(6):e1002130. doi: 10.1371/journal.pgen.1002130 (PMC3121749; doi:10.1371/journal.pgen.1002130)
Supplement: Text S1 — Supporting Experimental Procedures and Supporting References. Description of methods used for motif identification and statistical analysis of ChIP-Seq data and immunostaining of the protein markers of differentiation in cells with the associated supplementary references. (DOC) [file pgen.1002130.s010.doc]

**Text S1. Supporting Experimental Procedures.**

**ChIP-Seq Analysis**

Changes in the level of pSmad2 enrichment over genomic regions could be due to technical differences in sequencing efficiency of GC and AT rich regions rather than biological changes affecting transcription factor binding. To account for this, all ChIP-Seq libraries were sequenced to a comparable depth of 10 to 13 million unique tags on the Genome Analyzer (Illumina). Variations in the ratio of specific ChIP to input DNA library size in each condition were small in the range of 0.9 to 1.1. Normalized ChIP-Seq peak calling was carried out using the MACS algorithm [68] with a p-value cutoff of 1.0e-5 and 200bp extended tag length. The number of peaks in each condition containing overlaps (between the beginning and end of MACS peak coordinates) was counted to define common and unique peaks in the Venn diagram analysis (Fig 4A). All sequences were aligned and visualized on the UCSC Genome Browser on the mouse July 2007 (NCBI37/mm9) assembly (http://genome.ucsc.edu/), [69,70].

**Motif Analysis**

The central summit positions of ChIP-Seq peaks were used for downstream motif analysis. To calculate the frequency distribution of CAGA and CAGAC motifs in pSmad2 ChIP-Seq peaks, 1kb genomic sequences centered on the ChIP-seq binding peaks (+/-500bp) in each condition were extracted from the UCSC Genome browser. Subsequently, the exact matches of all possible 256 permutations of 4-mers, all 1024 permutations of 5-mers in these genomic intervals, as well as matches (with p<1e-05) of known Smad motifs from the TRANSFAC database (PWM SMAD_Q6 and SMAD_Q6_01) were counted using custom scripts. Motifs for the most highly enriched consensus pSmad2 binding sequences were identified using Weeder (http://159.149.109.9:8080/weederweb2006/), [71] and MEME (http://meme.nbcr.net/), [72] while co-motifs were identified by CEAS (http://ceas.cbi.pku.edu.cn/) and MotifEnrich (http://compbio.ddns.comp.nus.edu.sg/~chipseq/MotifEnrich/motifenrichinput.php), (Chang et al., manuscript in preparation). Comparison of *de novo* motifs to TRANSFAC and JASPAR PWM was done using the STAMP software (http://www.benoslab.pitt.edu/stamp/), [73].

**Statistical Analysis**

For pie chart statistics (Fig S4), all Venn diagram (Fig 4A) ChIP-Seq peak coordinates were used to obtain a non-redundant set of 12979 pSmad2 binding coordinates. A filter was applied to isolate 8635 peaks that were significantly above background consisting of at least 13 overlapping sequencing tags in any treatment. These peaks were classified into different modes of response to the 3 signaling conditions where there was at least a 1.25-fold change in pSmad2 enrichment in at least 1 condition compared to the others. The peaks were further mapped to within +/-50kb of all RefSeq mouse genes (mm9 assembly) to determine the types and distribution of pSmad2 binding at the gene level.

**Immunostaining**

Cells were fixed for 30 minutes in 4%PFA/PBS followed by 3 washes in 0.1% Triton X/PBS and permeabilized overnight in the last wash at 4°C. The cells were blocked with 1mg/ml BSA in 0.1% Tween-20/PBS (PBS-T) for 1 hour at 4°C then incubated with primary antibodies against rabbit anti-Mixl (Abcam), mouse anti-Lim1 (R&D Systems), goat anti-Oct4 (Santa Cruz Biotechnology), mouse anti-SSEA-1 (Millipore), rabbit anti-Hand1 (Santa Cruz Biotechnology) and mouse anti-P-cad (Thermo Scientific) at 1:100 dilution in 1mg/ml BSA PBS-T overnight at 4°C. The cells were washed 3 times with PBS-T followed by incubation for 30 minutes in 10μg/ml Hoechst 33342 counterstain with secondary antibodies 1:1000 Alexa Fluor 488 and 546 highly cross-adsorbed goat anti-rabbit IgG (H+L), Alexa Fluor 488 F(ab')2 fragment of goat anti-mouse IgG (H+L), Alexa Fluor 546 highly cross-adsorbed goat anti-mouse IgG (H+L) and Alexa Fluor 546 donkey anti-goat IgG (H+L) (Invitrogen). The cells were washed 3 times with PBS-T prior to image acquisition on an Olympus IX71 inverted fluorescence microscope.

**Supporting References**

68. Zhang Y, Liu T, Meyer CA, Eeckhoute J, Johnson DS, et al. (2008) Model-based analysis of ChIP-Seq (MACS). Genome Biol 9: R137.

69. Kent WJ, Sugnet CW, Furey TS, Roskin KM, Pringle TH, et al. (2002) The human genome browser at UCSC. Genome Res 12: 996-1006.

70. Waterston RH, Lindblad-Toh K, Birney E, Rogers J, Abril JF, et al. (2002) Initial sequencing and comparative analysis of the mouse genome. Nature 420: 520-562.

71. Pavesi G, Mereghetti P, Zambelli F, Stefani M, Mauri G, et al. (2006) MoD Tools: regulatory motif discovery in nucleotide sequences from co-regulated or homologous genes. Nucleic Acids Res 34: W566-570.

72. Bailey TL, Elkan C (1994) Fitting a mixture model by expectation maximization to discover motifs in biopolymers. Proc Int Conf Intell Syst Mol Biol 2: 28-36.

73. Mahony S, Benos PV (2007) STAMP: a web tool for exploring DNA-binding motif similarities. Nucleic Acids Res 35: W253-258.
